# Supplementary material for: Demystifying Cassiopea species identity in the Florida Keys: Cassiopea xamachana and Cassiopea andromeda coexist in shallow waters
Source: PLoS One. 2023 Mar 29;18(3):e0283441. doi: 10.1371/journal.pone.0283441 (PMC10058153; doi:10.1371/journal.pone.0283441)
Supplement: S1 File — (PDF) [file pone.0283441.s001.pdf]

Supplementary Materials

S1 Fig. *28S* tree with all *C. frondosa, C. andromeda and C. ornata* sequences from Genbank. KY611002-4 are likely misidentified *C. xamachana*. Support is organized as SH-aLRT/aBayes/bootstrap.

S2 Fig. Preserved specimens. Separated by species with whole body (row 1), rhopalium (row 2) and bell images (row 3) for each individual.

S1 Table. Specimen collection list. Specimen number is provided in first column, location is provided with approximate latitude (lat) and longitude (long). Salinity (in ppt), pH (to the nearest 0.1), surface temperature at the site in Celcius (Temp), date and time of collection, accession numbers for genes sequenced, whether a complete specimen was retained (Preserved) are reported for each medusa. Additionally, diameter of the medusa and species designation (CX for *C. xamachana* and CA for *C. andromeda*) are reported.

S2 Table: Sequences used for *28S* tree. All species IDs are as presented in GenBank. For individuals collected in this study, specimen ID is included in location in parentheses.

S1 Fig. ***28S*** **tree with all** ***C. frondosa, C. andromeda and C. ornata*** **sequences from Genbank.** KY611002-4 are likely misidentified *C. xamachana.* Support is organized as SH-aLRT/aBayes/bootstrap.

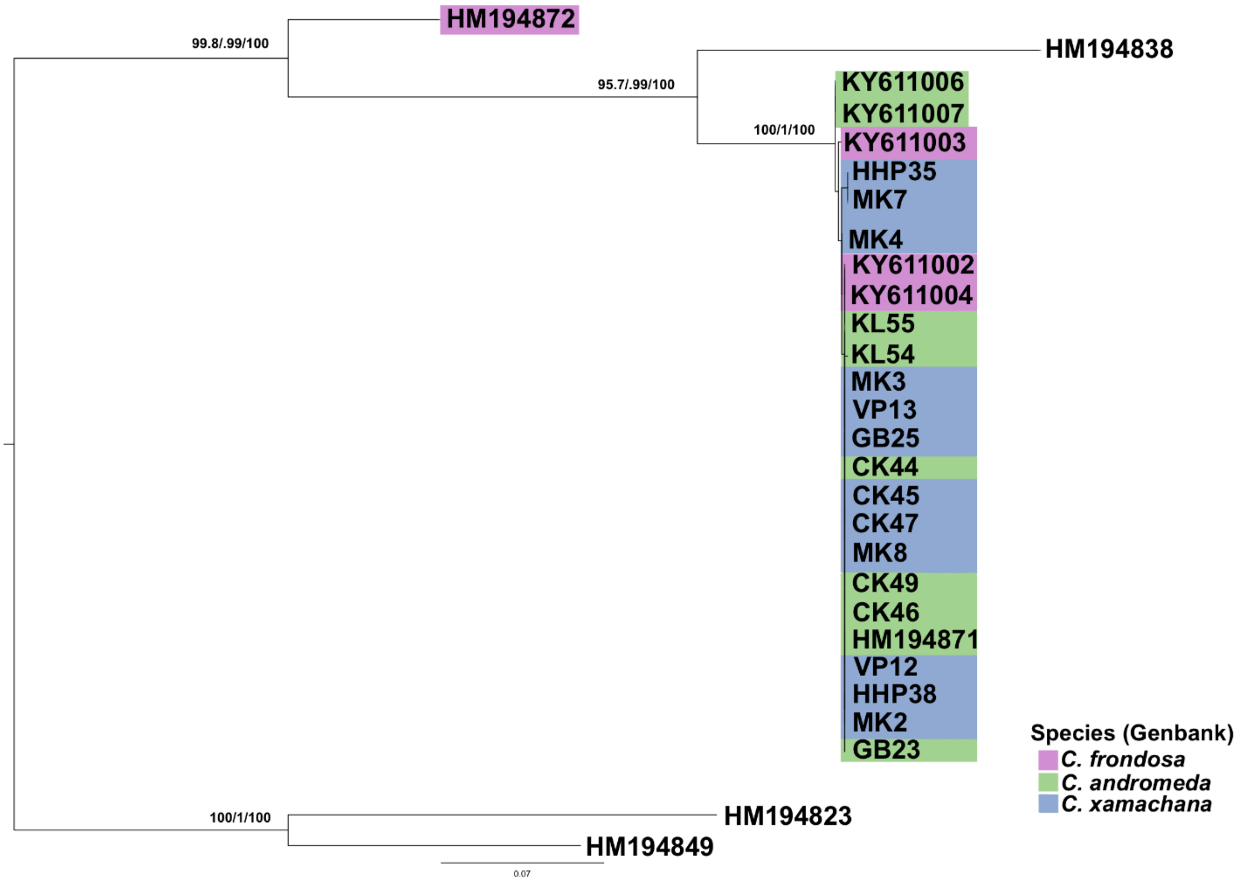

S2 Fig. Preserved specimens. Separated by species with whole body (row 1), rhopalium (row 2) and bell images (row 3) for each individual.

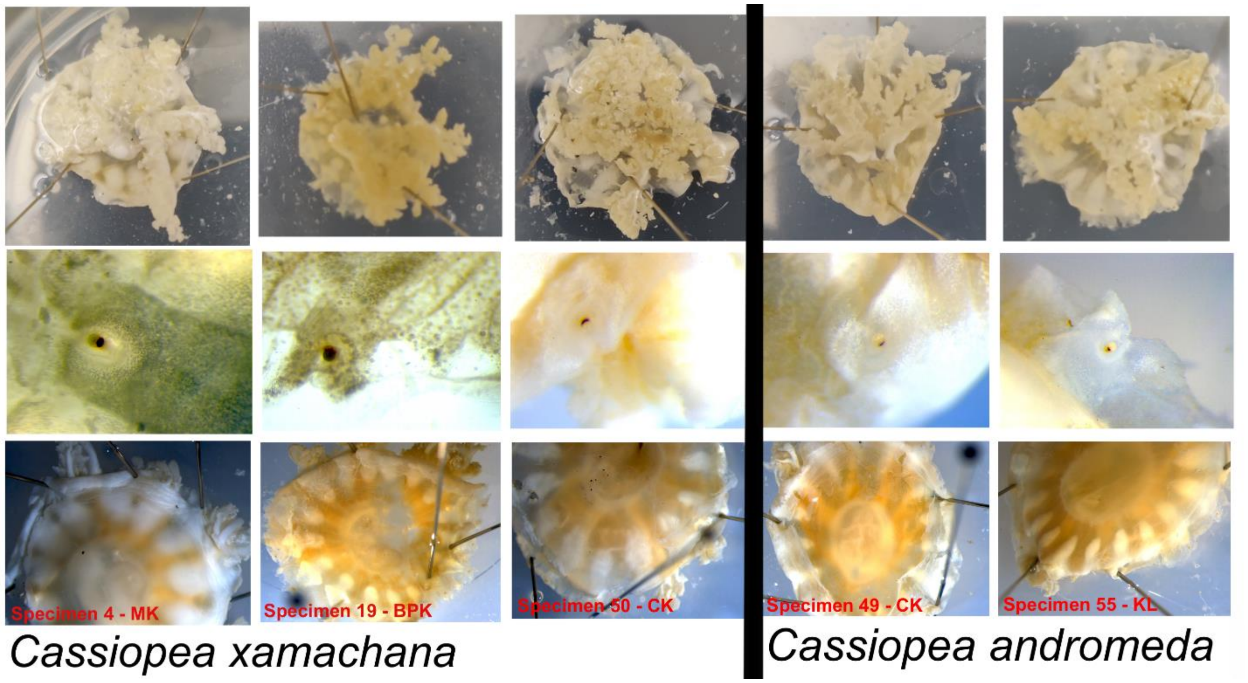

S1 Table. Specimen collection list. Specimen number is provided in first column, location is provided with approximate latitude (lat) and longitude (long). Salinity (in ppt), pH (to the nearest 0.1), surface temperature at the site in Celcius (Temp), date and time of collection, accession numbers for genes sequenced, whether a complete specimen was retained (Preserved) are reported for each medusa. Additionally, diameter of the medusa and species designation (CX for *C. xamachana* and CA for *C. andromeda*) are reported.

**Specimen**	**Lat**	**Long**	**Salinity (ppt)**	**pH**	**Temp (C)**	**Accession Numbers**	**Date**	**Pres-erved**	**Species**	**Time**	**Diameter (cm)**
1	24.69396	-81.0980507	31	8.1	35.2	*COI*: OP503313	11.8.2021	N	CX	12:03	6.13
2	24.69396	-81.0980507	31	8.1	35.2	*COI*: OP503314<br>*16S*: OP503902<br>*28S*: OP738399	11.8.2021	N	CX	12:19	8.527
3	24.69396	-81.0980507	30	8.3	35.7	*COI*: OP503315<br>*28S*: OP738400	11.8.2021	N	CX	16:52	4.288
4	24.69396	-81.0980507	30	8.3	35.7	*COI*: OP503354<br>*16S*: OP503903<br>*28S*: OP738413	11.8.2021	Y	CX	NR	1.9
5	24.69396	-81.0980507	30	8.3	35.7	*COI*: OP503316<br>*16S*: OP503904	11.8.2021	N	CX	17:19	6.951
6	24.69396	-81.0980507	30	8.3	35.7	*COI*: OP503355<br>*16S*: OP503905	11.8.2021	N	CX	17:32	8.898
7	24.69396	-81.0980507	30	8.3	35.7	*COI*: OP503356<br>*16S*: OP503906<br>*28S*: OP738415	11.8.2021	N	CX	17:55	6.984
8	24.69396	-81.0980507	30	8.3	35.7	*COI*: OP503357<br>*28S*: OP738401	11.8.2021	N	CX	18:14	8.5
9	24.69396	-81.0980507	30	8.3	35.7	*COI*: OP503358	11.8.	N	CX	NR	NR

							2021				
10	24.69396	-81.0980507	30	8.3	35.7	*COI*: OP503359<br>*16S*: OP503906	11.8.<br>2021	N	CX	NR	NR
11	24.682579<br>3	-81.2293673	37	8.3	31.8	*COI*: OP503317<br>*16S*: OP503907	13.8.<br>2021	N	CX	17:17	8.8
12	24.682579<br>3	-81.2293673	37	8.3	31.8	*COI*: OP503318<br>*16S*: OP503908<br>*28S*: OP738409	13.8.<br>2021	N	CX	17:54	15.66
13	24.682579<br>3	-81.2293673	37	8.3	31.8	*COI*: OP503319<br>*28S*: OP738402	13.8.<br>2021	N	CX	18:28	12.77<br>3
14	24.682579<br>3	-81.2293673	36	8	29.4	*COI*: OP503360	15.8.<br>2021	N	CX	9:32	9.348
15	24.682579<br>3	-81.2293673	36	8	29.4	*COI*: OP503361<br>*16S*: OP503909	15.8.<br>2021	N	CX	9:49	11.22<br>6
16	24.697873<br>1	-81.3572574	35	7.7	29.1	*COI*: OP503320	15.8.<br>2021	N	CX	12:18	5.601
17	24.697873<br>1	-81.3572574	35	7.7	29.1	*COI*: OP503321	15.8.<br>2021	N	CX	12:26	4.827
18	24.697873<br>1	-81.3572574	35	7.7	29.1	*COI*: OP503322<br>*16S*: OP503910	15.8.<br>2021	N	CX	12:40	5.716
19	24.697873<br>1	-81.3572574	35	7.7	29.1	*COI*: OP503362<br>*16S*: OP503911	15.8.<br>2021	Y	CX	NR	1.5
20	24.697873<br>1	-81.3572574	35	7.7	29.1	*COI*: OP503363	15.8.<br>2021	N	CX	NR	NR
21	24.697873<br>1	-81.3572574	35	7.7	29.1	*COI*: OP503323	15.8.<br>2021	N	CX	13:09	4.694
22	24.697873<br>1	-81.3572574	35	7.7	29.1	*COI*: OP503324<br>*16S*: OP503912	15.8.<br>2021	N	CX	13:18	4.743
23	24.561526	-81.7881727	36	8.2	30.4	*COI*: OP503325	15.8.<br>2021	N	CA	16:30	5.609

						16S: OP503913<br>*28S*: OP738412					
24	24.561526	-81.7881727	36	8.2	30.4	*COI*: OP503326<br>*16S*: OP503914	15.8.<br>2021	N	CX	16:36	5.283
25	24.561526	-81.7881727	36	8.2	30.4	*COI*: OP503327<br>*16S*: OP503915<br>*28S*: OP738403	15.8.<br>2021	N	CX	16:48	9.306
26	24.561526	-81.7881727	36	8.2	30.4	*COI*: OP503328<br>*16S*: OP503916	15.8.<br>2021	N	CX	16:52	8.816
27	24.561526	-81.7881727	36	8.2	30.4	*COI*: OP503329<br>*16S*: OP503917	15.8.<br>2021	N	CX	16:59	5.025
28	24.561526	-81.7881727	36	8.2	30.4	*COI*: OP503330<br>*16S*: OP503918	15.8.<br>2021	N	CX	17:06	5.264
29	24.561526	-81.7881727	36	8.2	30.4	*COI*: OP503331<br>*16S*: OP503919	15.8.<br>2021	N	CX	NR	NR
30	24.561526	-81.7881727	36	8.2	30.4	*COI*: OP503332<br>*16S*: OP503920	15.8.<br>2021	N	CX	17:15	7.184
31	24.561526	-81.7881727	36	8.2	30.4	*COI*: OP503333<br>*16S*: OP503921	15.8.<br>2021	N	CX	17:22	6.482
32	24.561526	-81.7881727	36	8.2	30.4	*COI*: OP503364<br>*16S*: OP503922	15.8.<br>2021	N	CX	NR	NR
33	25.023378<br>3	-80.4940316	31	8.1	33.3	*COI*: OP503334<br>*16S*: OP503922	16.8.<br>2021	N	CX	11:22	12.34
34	25.023378<br>3	-80.4940316	31	8.1	33.3	*COI*: OP503335<br>*16S*: OP503924	16.8.<br>2021	N	CX	11:32	17.90<br>4
35	25.023378<br>3	-80.4940316	31	8.1	33.3	*COI*: OP503336<br>*16S*: OP503925<br>*28S*: OP738416	16.8.<br>2021	N	CX	11:47	6.996

36	25.023378 3	-80.4940316	31	8.1	33.3	*COI*: OP503337 *16S*: OP503926	16.8. 2021	N	CX	12:06	15.38 8
37	25.023378 3	-80.4940316	31	8.1	33.3	*COI*: OP503338 *16S*: OP503927	16.8. 2021	N	CX	12:41	10.23 7
38	25.023378 3	-80.4940316	31	8.1	33.3	*COI*: OP503339 *16S*: OP503928 *28S*: OP738410	16.8. 2021	N	CX	12:56	10.7
39	24.858215 7	-80.7267927	35	8.1	33.0	*COI*: OP503340	16.8. 2021	N	CX	17:21	8.096
40	24.858215 7	-80.7267927	35	8.1	33.0	*COI*: OP503341 *16S*: OP503929	16.8. 2021	N	CX	17:31	14.72
41	24.677537 8	-81.4991819	36	8.4	32.6	*COI*: OP503342 *16S*: OP503930	17.8. 2021	N	CX	10:31	6.169
42	24.677537 8	-81.4991819	36	8.4	32.6	*COI*: OP503343 *16S*: OP503931	17.8. 2021	N	CX	10:45	6.207
43	24.677537 8	-81.4991819	36	8.4	32.6	*COI*: OP503344	17.8. 2021	N	CX	10:59	8.338
44	24.677537 8	-81.4991819	36	8.4	32.6	*COI*: OP503345 *16S*: OP503932 *28S*: OP738404	17.8. 2021	N	CA	11:19	7.128
45	24.677537 8	-81.4991819	36	8.4	32.6	*COI*: OP503346 *28S*: OP738405	17.8. 2021	N	CX	11:31	4.204
46	24.677537 8	-81.4991819	36	8.4	32.6	*COI*: OP503347 *16S*: OP503933 *28S*: OP738406	17.8. 2021	N	CA	11:48	6.417
47	24.677537 8	-81.4991819	36	8.4	32.6	*COI*: OP503348 *16S*: OP503934 *28S*: OP738407	17.8. 2021	N	CX	12:04	9.64
48	24.677537	-81.4991819	36	8.4	32.6	*COI*: OP503349	17.8.	N	CX	12:22	8.5

	8						2021				
49	24.6775378	-81.4991819	36	8.4	32.6	*COI*: OP503365<br>*16S*: OP503935<br>*28S*: OP738408	17.8.2021	Y	CA	NR	1.9
50	24.6775378	-81.4991819	36	8.4	32.6	*COI*: OP503366	17.8.2021	Y	CX	NR	1.8
51	25.0872104	-80.4415797	35	7.8	32.0	*COI*: OP503350<br>*16S*: OP503936	18.8.2021	N	CX	11:06	3.979
52	25.0872104	-80.4415797	35	7.8	32.0	*COI*: OP503351	18.8.2021	N	CX	NR	NR
53	25.0872104	-80.4415797	35	7.8	32.0	*COI*: OP503352<br>*16S*: OP503937	18.8.2021	N	CX	11:24	6.079
54	25.0872104	-80.4415797	35	7.8	32.0	*COI*: OP503353<br>*16S*: OP503938<br>*28S*: OP738411	18.8.2021	N	CA	11:34	5.441
55	25.0872104	-80.4415797	35	7.8	32.0	*COI*: OP503367<br>*16S*: OP503939<br>*28S*: OP738414	18.8.2021	Y	CA	NR	2.4

S2 Table: Sequences used for *28S* tree. All species IDs are as presented in GenBank. For individuals collected in this study, specimen ID is included in location in parentheses.

Species	Location	Accession Number	Source
C. frondosa	Key West, FL, USA	HM194872	Bayha 2010
C. ornata	Koror, Palau	HM194838	Bayha 2010
C. andromeda	Key West, FL, USA	HM194871	Bayha 2010
C. frondosa	Bocas del Toro, Panama	KY611002	Daglio et al. 2017
C. frondosa	Bocas del Toro, Panama	KY611003	Daglio et al. 2017
C. frondosa	Bocas del Toro, Panama	KY611004	Daglio et al. 2017

C. andromeda	Isla San Jose, Baja California, Mexico	KY611006	Daglio et al. 2017
C. andromeda	Isla San Jose, Baja California, Mexico	KY611007	Daglio et al. 2017
C. andromeda	(GB23) Garrison Bight, Key West, FL, USA	OP738412	This Study
C. andromeda	(CK44) Cudjoe Key, FL, USA	OP738404	This Study
C. andromeda	(CK46) Cudjoe Key, FL, USA	OP738406	This Study
C. andromeda	(CK49) Cudjoe Key, FL, USA	OP738408	This Study
C. andromeda	(KL54) Key Largo, FL, USA	OP738411	This Study
C. andromeda	(KL55) Key Largo, FL, USA	OP738414	This Study
C. xamachana	(MK2) Marathon, FL, USA	OP738399	This Study
C. xamachana	(MK3) Marathon, FL, USA	OP738400	This Study
C. xamachana	(MK4) Marathon, FL, USA	OP738413	This Study
C. xamachana	(MK7) Marathon, FL, USA	OP738415	This Study
C. xamachana	(MK8) Marathon, FL, USA	OP738401	This Study
C. xamachana	(VP12) Veterans Park, Bahia Honda, FL, USA	OP738409	This Study
C. xamachana	(VP13) Veterans Park, Bahia Honda, FL, USA	OP738402	This Study
C. xamachana	(GB25) Garrison Bight, Key West, FL, USA	OP738403	This Study
C. xamachana	(CK45) Cudjoe Key, FL, USA	OP738405	This Study
C. xamachana	(CK47) Cudjoe Key, FL, USA	OP738407	This Study
C. xamachana	(HHP35) Harry Harris Park, Tavernier, FL, USA	OP738416	This Study
C. xamachana	(HHP38) Harry Harris Park, Tavernier, FL, USA	OP738410	This Study
Versuriga anadyomene	Cemetery Reef, Palau	HM194823	Bayha 2010
Mastigias papua	Ongael Lake, Palau	HM194849	Bayha 2010
