# Supplementary material for: Demystifying Cassiopea species identity in the Florida Keys: Cassiopea xamachana and Cassiopea andromeda coexist in shallow waters
Source: PLoS One. 2023 Mar 29;18(3):e0283441. doi: 10.1371/journal.pone.0283441 (PMC10058153; doi:10.1371/journal.pone.0283441)
Supplement: Attachment — Submitted filename: PONE-D-22-31726_rev.pdf [file pone.0283441.s002.pdf]

PLOS ONE

Demystifying Cassiopea species identity in the Florida Keys: Cassiopea xamachana and Cassiopea andromeda coexist in shallow waters

--Manuscript Draft--

**Manuscript Number:**	PONE-D-22-31726
**Article Type:**	Research Article
**Full Title:**	Demystifying Cassiopea species identity in the Florida Keys: Cassiopea xamachana and Cassiopea andromeda coexist in shallow waters
**Short Title:**	Demystifying Cassiopea species identity in the Florida Keys
**Corresponding Author:**	Kaden Muffett<br>Texas A&M University at Galveston<br>Galveston, Texas UNITED STATES
**Keywords:**	Cassiopea, C. xamachana, C. andromeda, cryptic species
**Abstract:**	The phylogeny of the Upside-Down Jellyfish (Cassiopea spp.) has been upset multiple times in its history. This is especially true in the Florida Keys, where much of the Cassiopea stock for research and aquarium trade in the United States are collected. In August 2021, we collected 55 Cassiopea medusae at eight shallow water sites throughout the Florida Keys and sequenced COI, 16S, and 28S genes. Mitochondrial genes demonstrate that the shallow waters in Florida are inhabited by both C. xamachana and a non-native C. andromeda, identified in multispecies assemblages at least thrice. While C. xamachana were present at all sites, C. andromeda were present at only a minority of sites. This previously unknown multispecies assemblage is a likely root cause for the confusing and disputed species identities of Cassiopea in the Florida Key. It also serves as a cautionary note to all Cassiopea researchers to barcode their individuals regardless of the location in which they were collected.
**Order of Authors:**	Kaden Muffett
	Maria Pia Miglietta, PhD
**Additional Information:**	
**Question**	**Response**
**Financial Disclosure**<br><br>Enter a financial disclosure statement that describes the sources of funding for the work included in this submission. Review the submission guidelines for detailed requirements. View published research articles from *PLOS ONE* for specific examples.<br><br>This statement is required for submission and **will appear in the published article** if the submission is accepted. Please make sure it is accurate.	This work was supported by the National Science Foundation (NSF IOS #1936565) to author MPM. This work was also supported by the Texas Sea Grant to author KMM. The funders had no role in study design, data collection and analysis, decision to publish, or preparation of the manuscript.

Powered by Editorial Manager® and ProduXion Manager® from Aries Systems Corporation

**Unfunded studies**<br>Enter: *The author(s) received no specific funding for this work.*<br><br>**Funded studies**<br>Enter a statement with the following details:<br>• Initials of the authors who received each award<br>• Grant numbers awarded to each author<br>• The full name of each funder<br>• URL of each funder website<br>• Did the sponsors or funders play any role in the study design, data collection and analysis, decision to publish, or preparation of the manuscript?<br>• **NO** - Include this sentence at the end of your statement: *The funders had no role in study design, data collection and analysis, decision to publish, or preparation of the manuscript.*<br>• **YES** - Specify the role(s) played.<br><br>* typeset	
**Competing Interests**<br><br>Use the instructions below to enter a competing interest statement for this submission. On behalf of all authors, disclose any competing interests that could be perceived to bias this work—acknowledging all financial support and any other relevant financial or non-financial competing interests.<br><br>This statement is required for submission and **will appear in the published article** if the submission is accepted. Please make sure it is accurate and that any funding sources listed in your Funding Information later in the submission form are also declared in your Financial Disclosure statement.<br><br>View published research articles from *PLOS ONE* for specific examples.	The authors have declared that no competing interests exist.

**NO authors have competing interests**<br><br>Enter: *The authors have declared that no competing interests exist.*<br><br>**Authors with competing interests**<br><br>Enter competing interest details beginning with this statement:<br><br>*I have read the journal's policy and the authors of this manuscript have the following competing interests: [insert competing interests here]*<br><br>* typeset	
**Ethics Statement**<br><br>Enter an ethics statement for this submission. This statement is required if the study involved:<br><br>• Human participants<br>• Human specimens or tissue<br>• Vertebrate animals or cephalopods<br>• Vertebrate embryos or tissues<br>• Field research<br><br>Write "N/A" if the submission does not require an ethics statement.<br><br>General guidance is provided below. Consult the submission guidelines for detailed instructions. **Make sure that all information entered here is included in the Methods section of the manuscript.**	N/A

Powered by Editorial Manager® and ProduXion Manager® from Aries Systems Corporation

**Format for specific study types**<br><br>**Human Subject Research (involving human participants and/or tissue)**<br>• Give the name of the institutional review board or ethics committee that approved the study<br>• Include the approval number and/or a statement indicating approval of this research<br>• Indicate the form of consent obtained (written/oral) or the reason that consent was not obtained (e.g. the data were analyzed anonymously)<br><br>**Animal Research (involving vertebrate animals, embryos or tissues)**<br>• Provide the name of the Institutional Animal Care and Use Committee (IACUC) or other relevant ethics board that reviewed the study protocol, and indicate whether they approved this research or granted a formal waiver of ethical approval<br>• Include an approval number if one was obtained<br>• If the study involved *non-human primates*, add *additional details* about animal welfare and steps taken to ameliorate suffering<br>• If anesthesia, euthanasia, or any kind of animal sacrifice is part of the study, include briefly which substances and/or methods were applied<br><br>**Field Research**<br><br>Include the following details if this study involves the collection of plant, animal, or other materials from a natural setting:<br>• Field permit number<br>• Name of the institution or relevant body that granted permission	
**Data Availability**<br><br>Authors are required to make all data underlying the findings described fully available, without restriction, and from the time of publication. PLOS allows rare exceptions to address legal and ethical concerns. See the PLOS Data Policy and FAQ for detailed information.	Yes - all data are fully available without restriction

Powered by Editorial Manager® and ProduXion Manager® from Aries Systems Corporation

A Data Availability Statement describing where the data can be found is required at submission. Your answers to this question constitute the Data Availability Statement and **will be published in the article**, if accepted.<br><br>**Important:** Stating 'data available on request from the author' is not sufficient. If your data are only available upon request, select 'No' for the first question and explain your exceptional situation in the text box.<br><br>Do the authors confirm that all data underlying the findings described in their manuscript are fully available without restriction?	
**Describe where the data may be found in full sentences. If you are copying our sample text, replace any instances of XXX with the appropriate details.**<br><br>• If the data are **held or will be held in a public repository**, include URLs, accession numbers or DOIs. If this information will only be available after acceptance, indicate this by ticking the box below. For example: *All XXX files are available from the XXX database (accession number(s) XXX, XXX.*).<br>• If the data are all contained **within the manuscript and/or Supporting Information files**, enter the following: *All relevant data are within the manuscript and its Supporting Information files.*<br>• If neither of these applies but you are able to provide **details of access elsewhere**, with or without limitations, please do so. For example:<br><br>*Data cannot be shared publicly because of [XXX]. Data are available from the XXX Institutional Data Access / Ethics Committee (contact via XXX) for researchers who meet the criteria for access to confidential data.*<br><br>*The data underlying the results presented in the study are available from (include the name of the third party*	All relevant data and accession numbers are within the manuscript and its Supporting Information files.

and contact information or URL).<br>• This text is appropriate if the data are owned by a third party and authors do not have permission to share the data.<br><br>* typeset	
Additional data availability information:	

Powered by Editorial Manager® and ProduXion Manager® from Aries Systems Corporation

Manuscript

Click here to access/download;Manuscript;Dem_PLOS_11.17.22_justtext.doc

Demystifying *Cassiopea* species identity in the Florida Keys

Kaden Muffett* (orcid.org/0000-0002-3730-9832) and Maria Pia Miglietta (https://orcid.org/0000-0002-9458-593X)

Texas A&M University at Galveston, Galveston, TX, USA

*Corresponding Author: kmmuffett@tamu.edu

Abstract

The phylogeny of the Upside-Down Jellyfish (*Cassiopea* spp.) has been upset multiple times in its history. This is especially true in the Florida Keys, where much of the *Cassiopea* stock for research and aquarium trade in the United States are collected. In August 2021, we collected 55 *Cassiopea* medusae at eight shallow water sites throughout the Florida Keys and sequenced *COI*, *16S*, and *28S* genes. Mitochondrial genes demonstrate that the shallow waters in Florida are inhabited by both *C. xamachana* and a non-native *C. andromeda*, identified in multispecies assemblages at least thrice. While *C. xamachana* were present at all sites, *C. andromeda* were present at only a minority of sites. This previously unknown multispecies assemblage is a likely root cause for the confusing and disputed species identities of *Cassiopea* in the Florida Key. It also serves as a cautionary note to all *Cassiopea* researchers to barcode their individuals regardless of the location in which they were collected.

Keywords: *Cassiopea*, *C. xamachana*, *C. andromeda*, cryptic species

Introduction

Marine invertebrates have a wealth of cryptic lineages (1,2). Although our understanding of the diversity within cryptic marine taxa has grown, precise distributions of species are often challenging to assess with common sampling approaches. Scyphozoan phylogenies have frequently been sampled broadly but shallowly (3,4). In some pelagic systems, such as *Aurelia* or *Chrysaora*, this approach may be sufficient (5,6). However, in systems with a strong invasion potential and limited natural dispersion capabilities, like the genus *Cassiopea*, this shallow or single-site sampling may result in inadequate coverage to identify all species present.

As in other scyphozoan lineages, *Cassiopea* suffers from poor phylogenetic clarity. This is consequential because *C. xamachana*, and the genus *Cassiopea* more broadly, have become an emergent model system for research in symbiosis, behavior, and regeneration (7–11). Recent work has demonstrated that morphological, symbiosis and ecological differences exist between *Cassiopea* species (12,13), however, most non-taxonomic research is done on unspecified *Cassiopea* (14,15). This lack of clarity on the identity of the *Cassiopea* used in research, is problematic as it may lead to confounded, unreproducible, or less comparable experimental results.

High morphological heterogeneity within populations and apparent crypsis across species make *Cassiopea* difficult to identify (3,8,12). *Cassiopea* species identification and species boundaries are even more blurred in the Florida Keys, one of the main collection grounds for *Cassiopea* research in the United States (12). *Cassiopea* from Florida are often arbitrarily assigned one of three names: *C. xamachana*, *C. andromeda* or *C. frondosa*. *C. xamachana,* originally described in Jamaica by Bigelow 1892, theoretically represents the dominant ecotype in the Florida Keys and Caribbean (16). *C. xamachana*'s description is distinct from *C. andromeda* (Forskål, 1775), a Red Sea native and an invasive documented in Brazil, the Mediterranean, and Hawai'i (3,17,18). The separation of *C. xamachana* and *C. andromeda* is a point of contention, and the two species have been sometimes considered synonymous. When synonymized, *C. xamachana* was considered a population of introduced *C. andromeda* (8,19). This synonymization was originally a product of Gohar and Eisawy's efforts to reduce the many described *Cassiopea* species into only three species—*C. dieuphila* (species name not revisited), *C. andromeda* and *C. frondosa*-- based solely on one morphological character, the rhopaliar number (19). Rhopaliar number is now recognized as having high intraspecific variability and limited interspecific variability and thus not suitable for species delimitation (3,13). Four decades later, genetic sampling of *Cassiopea* by Holland et al. (2004) across the globe upended this oversimplistic morphological assessment and significantly expanded the known species diversity in the genus but found no evidence of a "*C. xamachana*" lineage. The individuals (n=4) collected from Bermuda and the Florida Keys were not distinct from other global *C. andromeda* populations (3). Since Holland et al. (2004), some of the *Cassiopea* species invalidated by Gohar and Eisawy (1960) were validated and characterized morphologically, however the *C. xamachana*/*C. andromeda* distinction was not reevaluated (13), as such we have not met the need for an updated and more in-depth phylogeny of the Florida Keys *Cassiopea*.

Here, using a concerted sampling of eight shallow water sites in the Florida Keys, and using mitochondrial and nuclear genes, as well as morphological data, we address the presence and the phylogenetic status of *C. xamachana* and *C. andromeda* in the Florida Keys.

Materials and Methods

Collection

In August 2021, 55 *Cassiopea* were collected by hand in 8 sites along the length of the Florida Keys. All sites were from near-shore water under 2 m depth (S1: site map, S2: specimen roster). *Cassiopea* were photographed and measured, and small tissue samples were preserved in ethanol (95%) at room temperature. Six small individuals were fully preserved in 95% EtOH for morphological analysis (Table S2).

DNA sequencing

DNA extractions from the 55 specimens were performed according to a salting out protocol (see full protocol in (20)). Mitochondrial Cytochrome c oxidate subunit I (*COI*) and *16S* ribosomal RNA (*16S*) were amplified using *Cassiopea*-specific protocols and primers (13). Nuclear *28S* was amplified using the *Cassiopea*-specific primers and protocols in Daglio et al. 2017. All products were purified using ExoSap and sanger sequenced at Texas A&M-Corpus Christi Genomics Core (Corpus Christi, Texas) or GeneWiz Azenta (Plainsfield, New Jersey). Fifty-five 514 bp segments of *COI*, 34 575 bp segments of *16S* and 18 822 bp segments of *28S* were assembled on Geneious and checked by eye.

Data analysis

C. xamachana and *C. andromeda* Clade Validation Dataset

Newly produced *COI* and *16S* sequences (55 and 38 sequences respectively) were aligned using Geneious 2022.2.2 (https://www.geneious.com) to *COI* and *16S* sequences from the *C. xamachana* and *C. andromeda* published genomes. Provisional sequence identity was assigned through agreement >98% to either genome. All sequences were uploaded to Genbank (see Table S2 for complete list of accession numbers).

Combined *COI* and *16S Cassiopea* Phylogeny Dataset

A subset of 11 representative *COI* sequences from individuals collected in this study (see Table 1 and S2) were aligned with *Cassiopea COI* GenBank sequences from Holland et al. 2004, Morandini et al. 2016, Daglio et al. 2017, Abboud et al. 2018 and Gamero-Mora et al. 2022 (see table) using MAFFT 7 (L-INS-i) (21). The final *COI* dataset was composed of 89 sequences, 11 of which were produced here, all trimmed to 514bp. The remaining 78 GenBank sequences belonged to the following species: *C. andromeda*, *C. xamachana*, *C. ornata*, *C.* sp. 1, *C.* sp. 2, *C.* sp. 3, *C. culionensis*, *C. mayeri*, *C. frondosa*, and outgroups *Mastigias papua* and *Versuriga anadyomene*.

Table 1: COI and 16S sequences used for tree generation, with both original identity from source and identity post-analysis. Isolates with the name *C. andromeda** were collected as *C.*

xamachana then redefined as *C. andromeda* in Holland et al. 2004 but as of this writing appear in GenBank as *C. xamachana*. In used in column: * denotes COI alignment, ^ denotes 16S alignment, + denotes COI haplotype network, # denotes 16S haplotype network.

Species	Reported species upon sequence publication	Locality	COI Accession	16S Accession	Dataset Used in	Source
C. andromeda	-	Cudjoe Key, FL, USA	OP503345	OP503932	*^+#	This study
C. andromeda	-	Key Largo, FL, USA	OP503353	OP503938	*^+#	This study
C. andromeda	-	Key West, FL, USA	OP503325	OP503913	*^+#	This study
C. andromeda	C. andromeda	Hilton lagoon, Waikiki, leeward O'ahu, Hawai'i, USA	AF231109	-	*+	Holland et al. 2004
C. andromeda	C. andromeda	Hilton lagoon, Waikiki, leeward O'ahu, Hawai'i, USA	AY319448	-	*+	Holland et al. 2004
C. andromeda	C. andromeda	Hilton lagoon, Waikiki, leeward O'ahu, Hawai'i, USA	AY319449	-	*+	Holland et al. 2004
C. andromeda	C. andromeda	Hilton lagoon, Waikiki, leeward O'ahu, Hawai'i, USA	AY319450	-	*+	Holland et al. 2004
C. andromeda	C. andromeda	Kainaone fish pond, Moloka'i, Hawai'i, USA	AY319453	-	*+	Holland et al. 2004
C. andromeda	C. andromeda	Kainaone fish pond, Moloka'i, Hawai'i, USA	AY319454	-	*+	Holland et al. 2004
C. andromeda	C. andromeda	El Ghardaqa, Egypt	AY319458	-	*+	Holland et al. 2004
C. andromeda	C. andromeda	French Polynesia	JN700934	JN700934	*^+#	Kayal et al. 2013
C. andromeda	C. andromeda	Brazil	KC464458	-	*+	Morandini et al. 2017
C. andromeda	C. andromeda	Isla San José, Baja California Sur, Mexico	KY610551	KY610609	*^+#	Daglio et al. 2017

C. andromeda	C. andromeda	Isla San José, Baja California Sur, Mexico	KY610552	-	*+	Daglio et al. 2017
C. andromeda	C. andromeda	Isla San José, Baja California Sur, Mexico	KY610553	-	*+	Daglio et al. 2017
C. andromeda	C. andromeda	Isla San José, Baja California Sur, Mexico	KY610554	-	*+	Daglio et al. 2017
C. andromeda	C. andromeda	Isla San José, Baja California Sur, Mexico	KY610555	-	*+	Daglio et al. 2017
C. andromeda	C. andromeda	Isla San José, Baja California Sur, Mexico	KY610556	-	*+	Daglio et al. 2017
C. andromeda	-	Key Largo, FL, USA	OP503367	OP503939	*^+#	This study
C. andromeda	C. sp.	Walsingham Pond, Hamilton, Bermuda	MF742175	-	*+	Abboud et al. 2018
C. andromeda	C. sp.	Mo'orea, Windward Islands, French Polynesia	MF742213	-	*+	Abboud et al. 2018
C. andromeda	C. sp.	Mo'orea, Windward Islands, French Polynesia	MF742214	-	*+	Abboud et al. 2018
C. andromeda	C. sp.	Mo'orea, Windward Islands, French Polynesia	MF742215	-	*+	Abboud et al. 2018
C. andromeda	*C. andromeda**	Walsingham Pond, Bermuda	AY319463	-	*+	Holland et al. 2004
C. andromeda	*C. andromeda**	Richardson Bay, Bermuda	AY319464	-	*+	Holland et al. 2004
C. andromeda	*C. andromeda**	Richardson Bay, Bermuda	AY319465	-	*+	Holland et al. 2004
C. andromeda	*C. andromeda**	Walsingham Pond, Bermuda	AY319466	-	*+	Holland et al. 2004
C. andromeda	*C. andromeda**	Key Largo, Florida, USA	AY319468	-	*+	Holland et al. 2004
C. xamachana	*C. frondosa*	Bahia Delfines, Bocas del Toro, Panama	KY610557	-	*+	Daglio et al. 2017

C. xamachana	*C. frondosa*	Bahia Delfines, Bocas del Toro, Panama	KY610558	-	*+	Daglio et al. 2017
C. xamachana	*C. frondosa*	Bahia Delfines, Bocas del Toro, Panama	KY610559	KY610614	*^+#	Daglio et al. 2017
C. xamachana	*C.* sp.	Key Largo, Florida, USA	MF742149	-	*+	Abboud et al. 2018
C. xamachana	*C.* sp.	Cassiopea Lake, Koror State, Palau	MF742166	-	*+	Abboud et al. 2018
C. xamachana	-	Marathon Key, FL,USA	OP503314	OP503902	*^+#	This study
C. xamachana	-	Tavernier, FL, USA	OP503334	OP503922	*^+#	This study
C. xamachana	-	Lobster Walk, Monroe County, FL, USA	OP503341	OP503929	*^+#	This study
C. xamachana	-	Cudjoe Key, FL, USA	OP503343	OP503931	*^+#	This study
C. xamachana	-	Big Pine Key, FL, USA	OP503317	OP503907	*^+#	This study
C. xamachana	-	Big Pine Key, FL, USA	OP503320	-	*+	This study
C. xamachana	-	Key West, FL, USA	OP503326	OP503914	*^+#	This study
C. xamachana	*C. xamachana*	Panama	JN700936	JN700936	*^+#	Kayal et al 2013
C. xamachana	*C. xamachana*	Bahia Delfines, Bocas del Toro, Panama	KY610560	-	*	Daglio et al. 2017
C. xamachana	*C. xamachana*	Bahia Delfines, Bocas del Toro, Panama	KY610561	-	*	Daglio et al. 2017
C. xamachana	*C. xamachana*	Bahia Delfines, Bocas del Toro, Panama	KY610562	-	*	Daglio et al. 2017
C. culionensis	*C. culionensis*	Monterey Bay Aquarium, USA	KF683387	-	*	Mellas et al 2014
C. culionensis	*C. culionensis*	Philippines	MW160923	MW164879	*^	Gamero-Mora 2022
C. culionensis	*C. culionensis*	Philippines	MW160930	MW164886	*^	Gamero-Mora

						2022
C. frondosa	*C. frondosa*	Key Largo, Florida, USA	AY319467	KY610617	*^	Holland et al. 2004 and Daglio et al. 2017
C. frondosa	*C. frondosa*	San Blas Islands, Panama	AY319469	-	*	Holland et al. 2004
C. frondosa	*C. frondosa*	San Blas Islands, Panama	AY319470	-	*	Holland et al. 2004
C. mayeri	*C. mayeri*	Japan	MW160931	MW164859	*^	Gamero-Mora 2022
C. mayeri	*C. mayeri*	Philippines	MW160934	MW164863	*^	Gamero-Mora 2022
C. mayeri	*C.* sp.	Sorido Bay, Kri, Papua	MF742205	-	*	Abboud et al. 2018
C. ornata	*C. ornata*	Short Drop Off, Palau	AY319456	-	*	Holland et al. 2004
C. ornata	*C. ornata*	Kakaban, Kalimantan, Indonesia	AY319472	AB720918	*^	Holland et al. 2004 and Gamero-Mora 2022
C. ornata	*C. ornata*	Kakaban, Kalimantan, Indonesia	AY319473	-	*	Holland et al. 2004
C. ornata	*C.* sp.	Risong Cove, Auluptagel Island, Koror State, Palau	MF742179	-	*	Abboud et al. 2018
C. ornata	*C.* sp.	Risong Cove, Auluptagel Island, Koror State, Palau	MF742193	-	*	Abboud et al. 2018
C. sp. *3*	*C.* sp. *3*	Kahuku windward, Oahu, Hawai’i, USA	AY319452	-	*	Holland et al. 2004

C. sp.	*C.* sp.	Coombabah Creek, Queensland, Australia	MF742133	-	*	Abboud et al. 2018
C. sp.	*C.* sp.	Coombabah Creek, Queensland, Australia	MF742135	-	*	Abboud et al. 2018
C. sp.	*C.* sp.	Kakaban, Berau, Kalimantan Timur, Indonesia	MF742139	-	*	Abboud et al. 2018
C. sp.	*C.* sp.	Kakaban, Berau, Kalimantan Timur, Indonesia	MF742140	-	*	Abboud et al. 2018
C. sp.	*C.* sp.	Kakaban, Berau, Kalimantan Timur, Indonesia	MF742141	-	*	Abboud et al. 2018
C. sp.	*C.* sp.	Kakaban, Berau, Kalimantan Timur, Indonesia	MF742142	-	*	Abboud et al. 2018
C. sp.	*C.* sp.	Haji Buang, Maratua, Berau, Kalimantan Timur, Indonesia	MF742143	-	*	Abboud et al. 2018
C. sp.	*C.* sp.	Haji Buang, Maratua, Berau, Kalimantan Timur, Indonesia	MF742148	-	*	Abboud et al. 2018
C. sp.	*C.* sp.	Danau Hidden Gam, Papua	MF742150	-	*	Abboud et al. 2018
C. sp.	*C.* sp.	Danau Hidden Gam, Papua	MF742151	-	*	Abboud et al. 2018
C. sp.	*C.* sp.	Mascot Channel, New Ireland, Papua New Guinea	MF742165	-	*	Abboud et al. 2018
C. sp.	*C.* sp.	Ongael Lake, Koror State, Palau	MF742183	-	*	Abboud et al. 2018
C. sp.	*C.* sp.	Lake Alexander, Northern Territory, Australia	MF742190	-	*	Abboud et al. 2018
C. sp.	*C.* sp.	Lake Alexander, Northern Territory, Australia	MF742191	-	*	Abboud et al. 2018

C. sp.	*C.* sp.	Lake Alexander, Northern Territory, Australia	MF742192	-	*	Abboud et al. 2018
C. sp.	*C.* sp.	Mascot Channel, New Ireland, Papua New Guinea	MF742209	-	*	Abboud et al. 2018
C. sp.	*C.* sp.	Mascot Channel, New Ireland, Papua New Guinea	MF742212	-	*	Abboud et al. 2018
C. sp. *1*	*C.* sp. *1*	Port Douglas, Queensland, Australia	AY319471	-	*	Holland et al. 2004
C. sp. *2*	*C.* sp.	Papua New Guinea	MF742198	-	*	Abboud et al. 2018
C. sp. *2*	*C.* sp.	Papua New Guinea	MF742199	-	*	Abboud et al. 2018
C. sp. *2*	*C.* sp. *2*	Observation Point, Papua New Guinea	AY319459	-	*	Holland et al. 2004
C. sp. *2*	*C.* sp. *2*	Observation Point, Papua New Guinea	AY319460	-	*	Holland et al. 2004
C. sp. *3*	*C.* sp.	Kagoshima Bay, Nagasuiro, Japan	MF742162	-	*	Abboud et al. 2018
C. sp. *3*	*C.* sp.	Nggatokae Mangroves, Western Solomon Islands	MF742189	-	*	Abboud et al. 2018
C. sp. *3*	*C.* sp. *3*	Emona, Papua New Guinea	AY319461	-	*	Holland et al. 2004
C. sp. *3*	*C.* sp. *3*	Emona, Papua New Guinea	AY319462	-	*	Holland et al. 2004
C. sp. *3*	*C.* sp. *3*	Wedding Chapel, windward O’ahu, Hawai’i, USA	AY331594	-	*	Holland et al. 2004
C. sp. *3*	*C.* sp. *3*	Kualoa Ranch, windward O’ahu, Hawai’i, USA	AY331595	-	*	Holland et al. 2004

C. xamachana	*C. xamachana*	eDNA Key Largo, FL, USA	-	MT709260	#	Ames et al. 2021
Unverified *Cassiopea* sequence	*C. andromeda*	eDNA Key Largo, FL, USA	-	MT709258	#	Ames et al. 2021
Mastigias papua	*Mastigias papua*	Mekeald Lake, Palau	KU901434	KY610621	*^	(Swift et al. 2016) and Daglio et al. 2017
Versuriga anadyomene	*Versuriga anadyomene*	Beibu Gulf, South China Sea	KX904853	KX904852	*^	(Sun et al. 2019)

16S sequences from 10 representative individuals across the Florida Keys (see Table 1) collected in this study were aligned with *Cassiopea 16S* GenBank sequences from Gamero-Mora 2022 and Daglio et al. 2017 and trimmed to 544 bp. The final *16S* dataset was composed of 22 sequences, ten from *C. xamachana* and *C. andromeda* from this study (same individuals as *COI* minus one) and 12 GenBank sequences belonging to *C. andromeda*, *C. xamachana*, *C. culionensis, C. mayeri*, *C. frondosa, C. ornata*, and outgroup taxa *Mastigias papua* and *Versuriga anadyomene.* *16S* sequences were aligned using MAFFT (E-INS-i).

Models for all *COI* codons and *16S* dataset were chosen by AICc from MEGA X 11 (22) model tester (*COI* codon position 1: TN93+I, *COI* codon position 2: TN93+I, *COI* codon position 3: TN93+G+I, *16S*: GTR+G+I). A dataset combining *COI* and *16S* was run in IQtree 1.6 under an ML framework with support from 1000 aLRT (approximate likelihood ratio test) and 1000 non-parametric bootstraps. Bayesian support was generated in BEAST 1.8 (23)with 10 million steps. All phylogenetic trees were edited with Figtree v1.4.4 (http://tree.bio.ed.ac.uk/software/figtree/).

28S Dataset

The *28S* dataset consisted of 28 sequences-- 18 newly produced *Cassiopea* sequences, 8 Genbank sequences of *C. frondosa, C. andromeda, C. ornata,* and two sequences belong to outgroups *Mastigias papua* and *Versuriga anadyomene* (see Fig S3 for all accession numbers). All 822 to 846 nucleotide *28S* sequences were aligned using MAFFT (G-INS-i). *28S* phylogenetic trees were run on IQtree under ML framework with support from aBayes, 1000 SH-aLRT (approximate likelihood ratio test) and 1000 non-parametric bootstraps under the Mega X model tester suggested model (TN93 + G) and edited in Figtree v1.4.

Haplotype networks

All 55 *COI* sequences from this study and 29 additional GenBank *COI* sequences belonging to *C. xamachana* and *C. andromeda* were used to generate a *COI* haplotype network. 38 *16S* sequences from this study and 7 additional GenBank *16S* sequences belonging to *C. xamachana* and *C. andromeda* were used to generate a *16S* haplotype network. Two additional *16S*

sequences produced from water samples in the Florida Keys for eDNA purposes (from Ames et al. 2021) were included in the *16S* haplotype map as these represented the only *Cassiopea 16S* data from the Florida Keys outside of the sequenced *C. xamachana* genome (see Table 1 for a complete list of sequences used). Haplotype networks were built using PopART v1.7 (24).

Results

Of the 55 *Cassiopea COI* sequences collected in the Florida Keys, 49 had a *Cassiopea xamachana* mitochondrial haplotype and six had a *C. andromeda* haplotype, as determined by agreement with published *C. xamachana* and *C. andromeda* mitogenomes. The divergence between the two species was approximately ~7%, as previously reported (13,25), with no intraspecific divergence within the *C. xamachana* or *C. andromeda* collected (Fig 1).

The *16S* and *COI* combined dataset, with sequences from GenBank, and rooted using sequences of *Mastigias papua* and *Versuriga anadyomene*, showed *C. xamachana* and *C. andromeda* as well supported sister taxa (posterior probability: 1, aLRT: 100%, bootstrap: 100%), and together as sister to a low supported clade that contains *C. ornata* from the Western Pacific , *C.* sp 1 from Australia, *C. culionensis*, *C.* sp 2 and *C. mayeri* from the Western Pacific. *C.* sp. 3 (from Papua New Guinea and Hawai'i) and *C. frondosa* are at the base of the tree (posterior probability: 1, aLRT: 99.5%, bootstrap: 100%) (Figure 1). Fourteen sampled individuals of undescribed *Cassiopea* from the Western Pacific (Abboud et al 2018) remain external to known *Cassiopea* taxa, and likely represent an additional undescribed species. Tree topology is in agreement with previous phylogenies (3,13).

The clade of *C. xamachana*, as defined by the published genome, is composed by sequences from the Florida Keys and Atlantic Panama, and one isolate from Palau (reported by Abboud et al. 2018). According to the analyzed dataset, except for the single Palau sequence, C. *xamachana* has not been identified outside of the Caribbean (Fig 3a). [illegible]*I* sequences identified as "*C. frondosa*" and collected in Panama (Atlantic), also fell within the *C. xamachana* clade. A third common species, *Cassiopea andromeda*, includes sequences from specimens collected in Hawai'i, Mexico, Bermuda, Brazil, Florida and the Red Sea (Fig 3a & 3b).

Cassiopea xamachana and *C. andromeda* had ~3.1% divergence in *16S* sequences, lower than that calculated for *COI*. The six *16S* sequences from collected *C. andromeda* showed no intraspecific diversity. The 32 *C. xamachana 16S* sequences showed low intraspecific diversity (d=0.0006) (Figure 2).

When considering the *COI* dataset used for haplotype network building, *Cassiopea andromeda* showed 11 total haplotypes and *C. xamachana* a single haplotype (Figure 3a). *Cassiopea xamachana* showed three *16S* haplotypes, and *C. andromeda* two, though only one was from specimens collected in this study (Figure 3b). The most common *C. xamachana 16S* haplotype was present in both the Keys and Panama. The second largest *16S* haplotype was present only in upper Keys sites, in conjunction with a single individual from Big Pine Key showing a single nucleotide change. In the *16S* haplotype network, there is one intermediate between *C. xamachana* and *C. andromeda* individuals, however this sequence was collected as

part of an eDNA project and likely does not represent a mitochondrial haplotype present in the system (26) .

The nuclear *28S* sequences (n=18) from all sampled *Cassiopea* showed polymorphism, and tree topology was incongruent with mitochondrial genes sequenced (Figure S4). Specifically, sequences belonging to specimens collected in this study identified as *C. xamachana* and *C. andromeda* showed no differentiation. Genbank sequences of *C. andromeda* from Baja California (Genbank Acc. KY611005-7) included gaps not found in the sequences from the Keys and were closely related to two GenBank sequences of "*C. frondosa*" from Panama (likely *C. xamachana*) (Genbank Acc. KY611002-3). *C. ornata* from Palau and true *C. frondosa* from Key West (GenBank Acc. HM194838 and HM194872) were divergent from the *C. xamachana*/*C. andromeda* clade and each other. Given the low mitochondrial divergence relative to a 10% benchmark, the *28S* results may indicate introgression and hybridization between *C. andromeda* and *C. xamachana*. However, given the limited *28S* coverage across *Cassiopea*, it is similarly likely that *28S* is not informative in distinguishing species within the genus.

Geographic distribution of *C. andromeda* and *C. xamachana* in the Florida Keys

We collected 55 samples in 8 localities along the Florida Keys (Figure S1). With 49/55 individuals, *C. xamachana* was more frequently found in samples (Fig 3b). *C. xamachana* was found in all sites and *C. andromeda* in three of eight sampling sites. In the three sites that hosted the two species, *C. xamachana* was more abundant than *C. andromeda* (proportion of *C. andromeda*: 3/10 on Cudjoe Key, 1/10 on Key West, 2/5 on Key Largo). Two of the five monospecific *C. xamachana* sites were shallow lagoons. The other two were low coverage tidal oceanic sites with low density and large individuals. The three sites of cohabitation were densely populated sites (>10 medusae/m^2) with calm water but direct oceanic exposure. Both the Key Largo site and the Key West site were at marinas.

C. andromeda have been found on both the Atlantic and Gulf sides of Key Largo, as well as on the Gulf side of Cudjoe Key and Key West. Although our sampling seems to indicate a reduced population of *C. andromeda* in the Middle Key, deeper sampling is needed to further support this hypothesis.

Size and Color

At each location, *C. andromeda* and *C. xamachana* presented the same color type and similar morphology (see Supplementary Materials), with no apparent character that could distinguish between the two species. Overall, individuals with the *C. andromeda* mitotype had somewhat smaller diameters (mean= 4.8 cm) than *C. xamachana* (mean= 7.8 cm) but not to a significant degree (ANOVA, $F(2,47) = 3.69$, $P = 0.061$). Two of the sites where *C. androme*[illegible] were found (Cudjoe Key and Key Largo Ocean Bay Marina) had primarily small individuals, and site was the most important factor in size determination (ANOVA, $F(1,47) = 5.08$, $P = 0.029$). As all specimens were preserved in ethanol as opposed to formalin, no in-depth comparative morphological analyses were performed. A general inspection showed that *C. andromeda* (n=2) and *C. xamachana* (n=3), when devoid of symbionts, had different bell

markings, however, with such a limited dataset, no conclusions could be drawn (Fig. S5). All specimen photographs are included in supplementary materials.

With regard to coloration, *Cassiopea* collected from this work were blue, white, purple, pink, brown, and green. Color profiles were generally consistent within sites but were highly variable between sites (Fig 4). This is a well-known phenomenon in *Cassiopea*, and the exact dynamics of color are poorly understood.

Discussion

Cassiopea xamachana, has been dramatically genetically undersampled in the Florida Keys relative to its use in research and laboratory work (11,14,27,28). A belief that *C. xamachana* was only a *C. andromeda* subpopulation likely contributed to this lack of focus on dense geographic sampling (3,6). Moreover, in the last 20 years *C. xamachana COI* isolates have occasionally been misidentified as *C. frondosa*, a very distantly related *Cassiopea*, further adding to the taxonomic confusion. Our data support the notion that *C. xamachana* and *C. andromeda* are two distinct species and are now both found in the Florida Keys. Although mitochondrial DNA shows these two species are reciprocally monophyletic clades, they are morphologically undistinguishable and they inhabit the same shallow water, sometimes coexisting side by side in the same location. 89% of our samples matched *C. xamachana*. *C. xamachana* was also sampled in 100% of sampling sites (8/8), while *C. andromeda* was sampled in 38% (3/8). We thus show that in our sampling effort *C. xamachana* is more abundant both in terms of number of jellyfish and locations where it is found. Our results also confirm recent eDNA analyses conducted in the same area that recorded *16S* residues of both species in the Keys (26). Despite having more representatives in this study, there was no diversity within the *Cassiopea xamachana COI* sequences and little diversity in their *16S* profiles. This may represent a higher degree of continuity across the Florida Keys and Panama than expected. Further study of the exact boundaries that impact *Cassiopea* genetic populations is needed, as it is clear that the degree of invasion demonstrated by the *C. andromeda* clade has not yet been demonstrated in other *Cassiopea* species.

While there has been no demonstrated divergence in behavior or tolerance to environmental factor between *C. xamachana* and *C. andromeda*, there has also been no study formally comparing them. Our data show that wild-caught *Cassiopea*, even from single locations present clear hazards for comparative analysis if not properly identified (4). This brings into foreground the inadvisability of treating results from Floridian samples and other locations as representatives of one clade without genetic evidence.

While the mitochondrial markers analyzed in this work present a clear picture of genetic separation between *C. xamachana* and *C. andromeda*, the nuclear marker (*28S*) does not. As *Cassiopea* has very few published *28S* sequences, some of which certainly suffer from the same issues of misidentification as the *COI* isolates, firm conclusions cannot be drawn as to the usefulness of *28S* for species delimitation. It is notable that all *28S* isolates from this study segment together regardless of mitotype, leaving the possibly that there is hybridization along the

range. In-depth study of a larger array of nuclear markers is needed to parse the hybridization potential or degree of *C. xamachana* and *C. andromeda* within the Florida Keys.

As only 11% of individuals found in the Keys in this sampling effort were *C. andromeda*, only relatively dense sampling (either large collections from multiple locations or environmental sequencing) could identify the mix. This raises concerns that other locations with *Cassiopea*, especially those with a paucity of sequences, may present the sort of cryptic or near-cryptic species assemblages already identified here in the Florida Keys, Hawai'i, the Philippines and Palau (3,6,13). Finally, in constructing the phylogeny, we found evidence of several instances of *Cassiopea* Genbank sequences attributed to the wrong species. Three *COI* sequences identified as *Cassiopea frondosa* were instead *C. xamachana*. Additionally, five sequences identified as *C. xamachana* sequences were instead *C. andromeda*, despite their originating text correctly identifying their species affinity (3). This accounts for five of sixteen total identified *C. andromeda* sequences. This indicates a general problem with GenBank sequences and is a result of the taxonomic confusion that has surrounded *Cassiopea* species.

Conclusion

Cassiopea in the Florida Keys has long been defined as two species, the deeper-water, distinctive *C. frondosa* and the shallow-water *C. xamachana* (12). In 1960 and again in 2004, *C. xamachana* was relegated to a junior synonym of *C. andromeda* (3,19). Using a phylogenetic approach, we show that *C. andromeda* and *C. xamachana* mitochondrial genotypes are both found in sympatry in the Florida Keys, showing no obvious morphological differences. We show that it is difficult to determine the species of *Cassiopea* collected in shallow water in the Keys without proper molecular barcoding. This is relevant because a wealth of research has been performed with various *Cassiopea* without a proper assessment of the species it was conducted on. We also found evidence that *Cassiopea* research has suffered from frequent species misidentification. This paper calls for deeper sampling of jellyfish assemblages within *Cassiopea* and other highly cryptic scyphozoan genera. It also indicated that a proper species identification that involves molecular barcoding is essential for any work on *Cassiopea*, especially from Florida. This even more crucial as *Cassiopea* continues to successfully establish itself as an emerging model system for physiological studies and as a proxy for investigations on zooxanthellae-Cnidaria interaction. Caution should be exercised in generalizing result from published studies that assumed *Cassiopea* identity without explicitly investigating species identification with molecular tools.

Compliance with Ethical Standards

The authors declare no conflict of interest. This grant was funded in part by Texas Sea Grant and by NSF IOS #1936565 to MPM.

Acknowledgments

We thank the NSF and NOAA Texas Sea Grant for funding this work.

References

1. Dawson MN, Jacobs DK. Molecular evidence for cryptic species of *Aurelia aurita* (Cnidaria, Scyphozoa). Biol Bull [Internet]. 2001 Feb [cited 2019 Nov 25];200(1):92–6. Available from: http://www.ncbi.nlm.nih.gov/pubmed/11249217

2. Cerca J, Meyer C, Purschke G, Struck TH. Delimitation of cryptic species drastically reduces the geographical ranges of marine interstitial ghost-worms (Stygocapitella; Annelida, Sedentaria). Mol Phylogenet Evol [Internet]. 2020;143(October 2019):106663. Available from: https://doi.org/10.1016/j.ympev.2019.106663

3. Holland BS, Dawson MN, Crow GL, Hofmann DK. Global phylogeography of *Cassiopea* (Scyphozoa: Rhizostomeae): Molecular evidence for cryptic species and multiple invasions of the Hawaiian Islands. Mar Biol. 2004;145(6):1119–28.

4. Gómez Daglio L, Dawson MN. Species richness of jellyfishes (Scyphozoa:Discomedusae) in the Tropical Eastern Pacific: Missed taxa, molecules, and morphology match in a biodiversity hotspot. Invertebr Syst. 2017;31(5):635–63.

5. Dawson MN, Martin LE. Geographic variation and ecological adaptation in *Aurelia* (Scyphozoa, Semaeostomeae): Some implications from molecular phylogenetics. Hydrobiologia. 2001;451:259–73.

6. Abboud SS, Daglio LG, Dawson MN. A global estimate of genetic and geographic differentiation in macromedusae-implications for identifying the causes of jellyfish blooms. Mar Ecol Prog Ser. 2018;591:199–216.

7. McGill CJ, Pomory CM. Effects of bleaching and nutrient supplementation on wet weight in the jellyfish *Cassiopea xamachana* (Bigelow) (Cnidaria: Scyphozoa). Mar Freshw Behav Physiol. 2008;41(3):179–89.

8. Ohdera AH, Abrams MJ, Ames CL, Baker DM, Suescún-Bolívar LP, Collins AG, et al. Upside-Down but Headed in the Right Direction: Review of the Highly Versatile *Cassiopea xamachana* System. Front Ecol Evol. 2018 Apr 9;6:1–15.

9. Freeman CJ, Stoner EW, Easson CG, Matterson KO, Baker DM. Symbiont carbon and nitrogen assimilation in the *Cassiopea-Symbiodinium* mutualism. Mar Ecol Prog Ser [Internet]. 2016 Feb 18 [cited 2022 Mar 31];544:281–6. Available from: https://www.int-res.com/abstracts/meps/v544/p281-286/

10. Klein SG, Pitt KA, Carroll AR. Surviving but not thriving: Inconsistent responses of zooxanthellate jellyfish polyps to ocean warming and future UV-B scenarios. Sci Rep. 2016 Jul 4;6.

11. Röthig T, Puntin G, Wong JCY, Burian A, McLeod W, Baker DM. Holobiont nitrogen control and its potential for eutrophication resistance in an obligate photosymbiotic jellyfish. Microbiome. 2021;9(1):1–16.

12. Fitt WK, Hofmann DK, Kemp DW, Ohdera AH. Different Physiology in the Jellyfish *Cassiopea xamachana* and *C. frondosa* in Florida Bay. Oceans. 2021;2(4):811–21.

13. Gamero-Mora E, Collins AG, Boco SR, Geson SM, Morandini AC. Revealing hidden diversity among upside-down jellyfishes (Cnidaria: Scyphozoa: Rhizostomeae: Invertebr Syst. 2022;36(1):63–89.

14. Klein SG, Pitt KA, Carroll AR. Reduced salinity increases susceptibility of zooxanthellate jellyfish to herbicide toxicity during a simulated rainfall event. Environ Pollut [Internet]. 2016;209:79–86. Available from: http://dx.doi.org/10.1016/j.envpol.2015.11.012

15. Ojimi MC, Isomura N, Hidaka M. Telomerase activity is not related to life history stage in the jellyfish *Cassiopea* sp. Comp Biochem Physiol - A Mol Integr Physiol [Internet]. 2009;152(2):240–4. Available from: http://dx.doi.org/10.1016/j.cbpa.2008.10.008

16. Bigelow R. The Anatomy and Development of *Cassiopea xamachana*. Bost Soc Nat Hist. 1900;387–91.

17. Morandini AC, Stampar SN, Maronna MM, Da Silveira FL. All non-indigenous species were introduced recently? the case study of *Cassiopea* (Cnidaria: Scyphozoa) in Brazilian waters. J Mar Biol Assoc United Kingdom. 2017;97(2):321–8.

18. Deidun A, Gauci A, Sciberras A, Piraino S. Back with a bang – an unexpected massive bloom of *Cassiopea andromeda* (Forskaal , 1775) in the Maltese Islands , nine years after its first appearance. 2018;7(4):399–404.

19. Gohar HAF, Eisawy AM. The biology of *Cassiopea andromeda* (from the Red Sea)(with a note on the species problem). Publ Mar Biol Station Al-Ghardaqa. 1960;11:5–42.

20. Frolova A, Muffett K, Miglietta MP. Multiple occurrences of *Callinectes sapidus* larvae on Gulf of Mexico *Chrysaora chesapeakei.* J Plankton Res [Internet]. 2022 Sep 19 [cited 2022 Oct 31]; Available from: https://academic.oup.com/plankt/advance-article/doi/10.1093/plankt/fbac053/6702819

21. Katoh K, Standley DM. MAFFT multiple sequence alignment software version 7: Improvements in performance and usability. Mol Biol Evol. 2013;30(4):772–80.

22. Tamura K, Stecher G, Kumar S. MEGA11: Molecular Evolutionary Genetics Analysis Version 11. Mol Biol Evol. 2021;38(7):3022–7.

23. Suchard MA, Lemey P, Baele G, Ayres DL, Drummond AJ, Rambaut A. Bayesian phylogenetic and phylodynamic data integration using BEAST 1.10. Virus Evol. 2018;4(1):1–5.

24. Leigh JW, Bryant D. POPART: Full-feature software for haplotype network construction. Methods Ecol Evol. 2015;6(9):1110–6.

25. Kayal E, Roure B, Philippe H, Collins AG, Lavrov D V. Cnidarian phylogenetic relationships as revealed by mitogenomics. BMC Evol Biol [Internet]. 2013 Jan 9 [cited 2020 Mar 5];13(1):5. Available from: http://bmcevolbiol.biomedcentral.com/articles/10.1186/1471-2148-13-5

26. Ames CL, Ohdera AH, Colston SM, Collins AG, Fitt WK, Morandini AC, et al. Fieldable Environmental DNA Sequencing to Assess Jellyfish Biodiversity in Nearshore Waters of the Florida Keys, United States. Front Mar Sci. 2021;8(April):1–17.

27. Ohdera A, Ames CL, Dikow RB, Kayal E, Chiodin M, Busby B, et al. Box, stalk[illegible] and upside-down? Draft genomes from diverse jellyfish (cnidaria, acraspeda) lineages: *Alatina alata* (cubozoa), *Calvadosia cruxmelitensis* (staurozoa), and *Cassiopea xamachana* (scyphozoa). Gigascience. 2019;8(7):1–15.

28. Niggl W, Wild C. Spatial distribution of the upside-down jellyfish *Cassiopea* sp. within fringing coral reef environments of the Northern Red Sea : implications for its life cycle. Helgol Mar Res. 2010;64:281–7.

29. Swift HF, Gómez Daglio L, Dawson MN. Three routes to crypsis: Stasis, convergence, and parallelism in the *Mastigias* species complex (Scyphozoa, Rhizostomeae). Mol Phylogenet Evol [Internet]. 2016;99:103–15. Available from: http://dx.doi.org/10.1016/j.ympev.2016.02.013

30. Sun T, Dong Z, Li Y. *Versuriga anadyomene*, a newly recorded scyphozoan jellyfish (Scyphozoa: Rhizostomae) in Chinese waters. J Oceanol Limnol. 2019;37(1):266–72.

<u>Statements and Declarations</u>

Funding: This work was supported by the National Science Foundation (NSF IOS #1936565) to author MPM. This work was also supported by the Texas Sea Grant to author KMM.

Competing interests: Author MPM and KMM declare they have no financial interests.

Author Contributions: Study design, material preparation, data collection and analysis were performed by KMM. The first draft of the manuscript was written by KMM. Funding and manuscript revisions were performed by MPM. All authors read and approved the final manuscript.

Data Availability: The sequences generated by or otherwise utilized by this study are available on GenBank, see specific identifiers in Table 1 and supplementary materials.

Ethics Approval: This work was not performed on vertebrates or protected species.

Click here to access/download;Figure;Dem_PLOS_11.17.2022_figs.docx

Figures (with captions)

Figure 1. ML COI + 16S tree with posterior probability, aLRT, and bootstrap supports. Individuals in grey were sequenced in this study. All accession numbers are for COI, see 16S accession numbers in Table 1. Asterisk (*) represents under .70 (PP) or 70 (aLRT and bootstrap) support. Species names are assigned in accordance with Gamero-Mora et al. 2022.

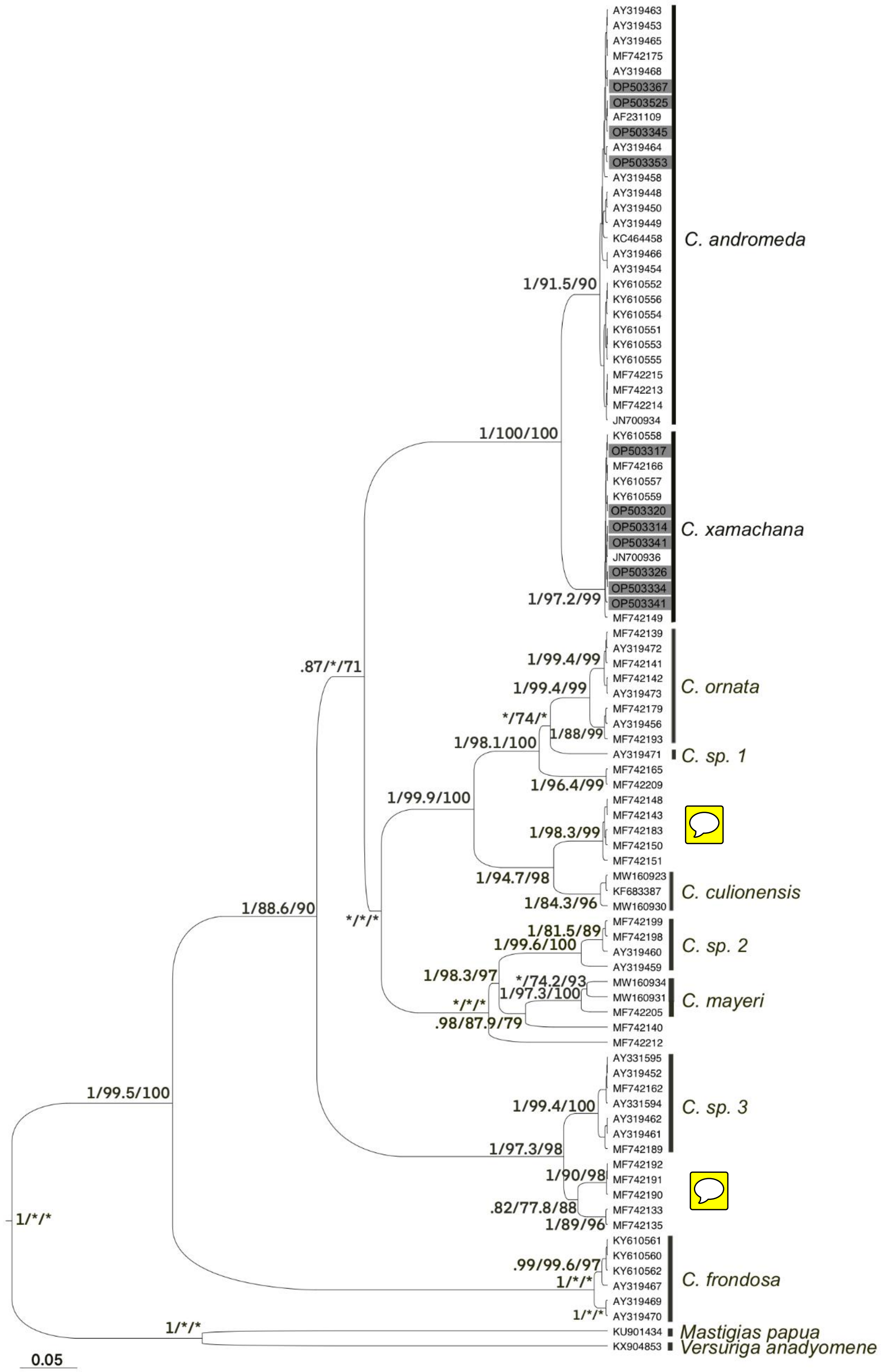

C. andromeda
C. xamachana
C. ornata
C. sp. 1
C. culionensis
C. sp. 2
C. mayeri
C. sp. 3
C. frondosa
Mastigias papua
Versuriga anadyomene
0.05

Figure 2. 16S haplotype network (right) and COI haplotype network (left) for *C. xamachana* and *C. andromeda*. One accession number that is consistent with the haplotype is displayed next to each group. Sequence MT709258***** is a product of eDNA work and may not represent a genuine haplotype.

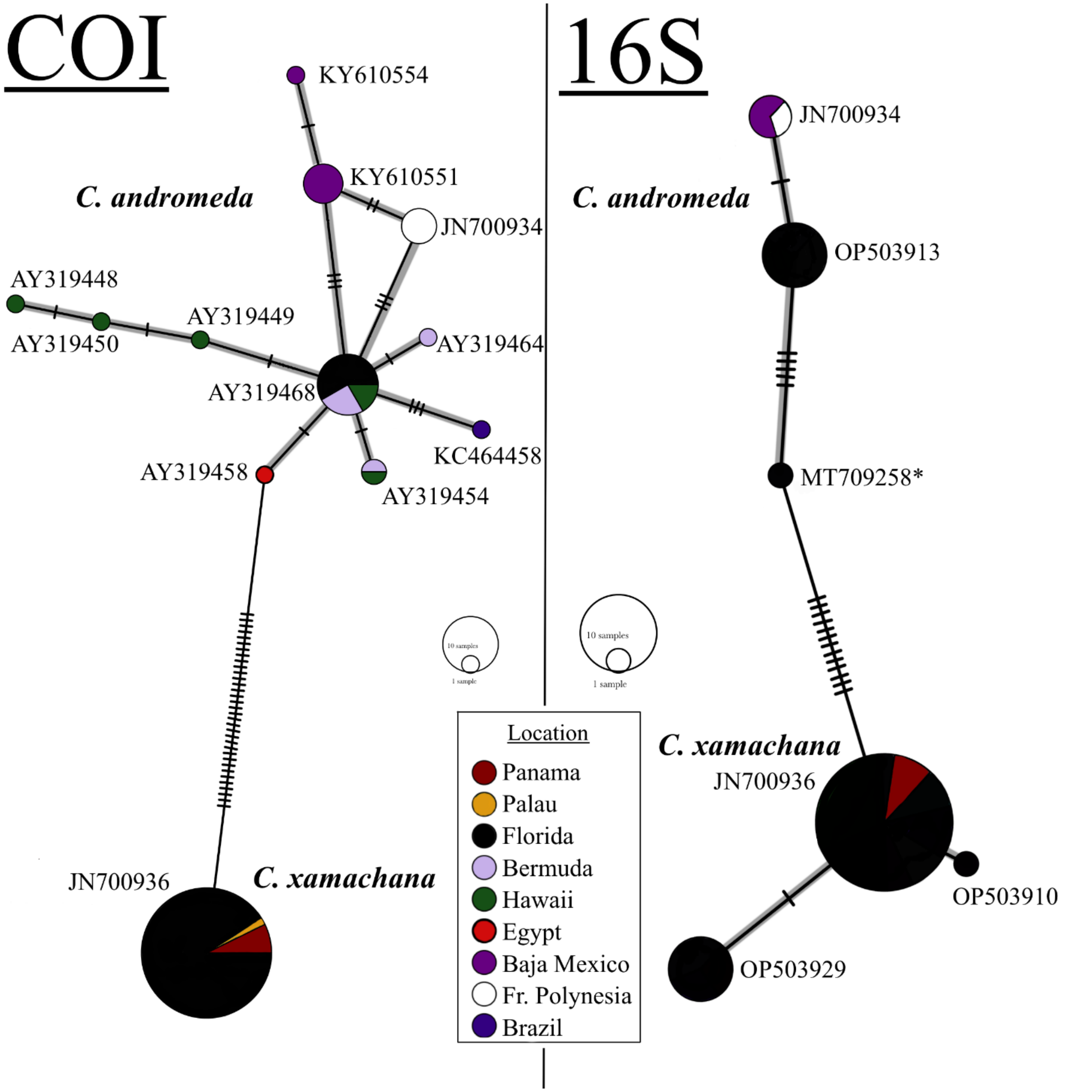

Figure 3. (a) *C. andromeda* (black) and *C. xamachana* (white) distributions from sequences published in relevant scyphozoan or *Cassiopea* specific phylogenies from 2004-2022 (Holland et al. 2004; Morandini et al. 2017; Abboud et al. 2018; Gamero-Mora et al. 2022). Locations with both recorded as black-and-white. Red square indicates Florida Keys. **(b)** *C. andromeda* (black) and *C. xamachana* (white) isolates from the Florida Keys from this study, pie chart indicates proportion of specimens and size of chart indicates number collected. The black dot with grey pie chart indicates the commonly visited Key Largo collection site at the Key Largo Marine Research Laboratory (KLMRL) at which both *C. andromeda* and *C. xamachana* isolates have been found.

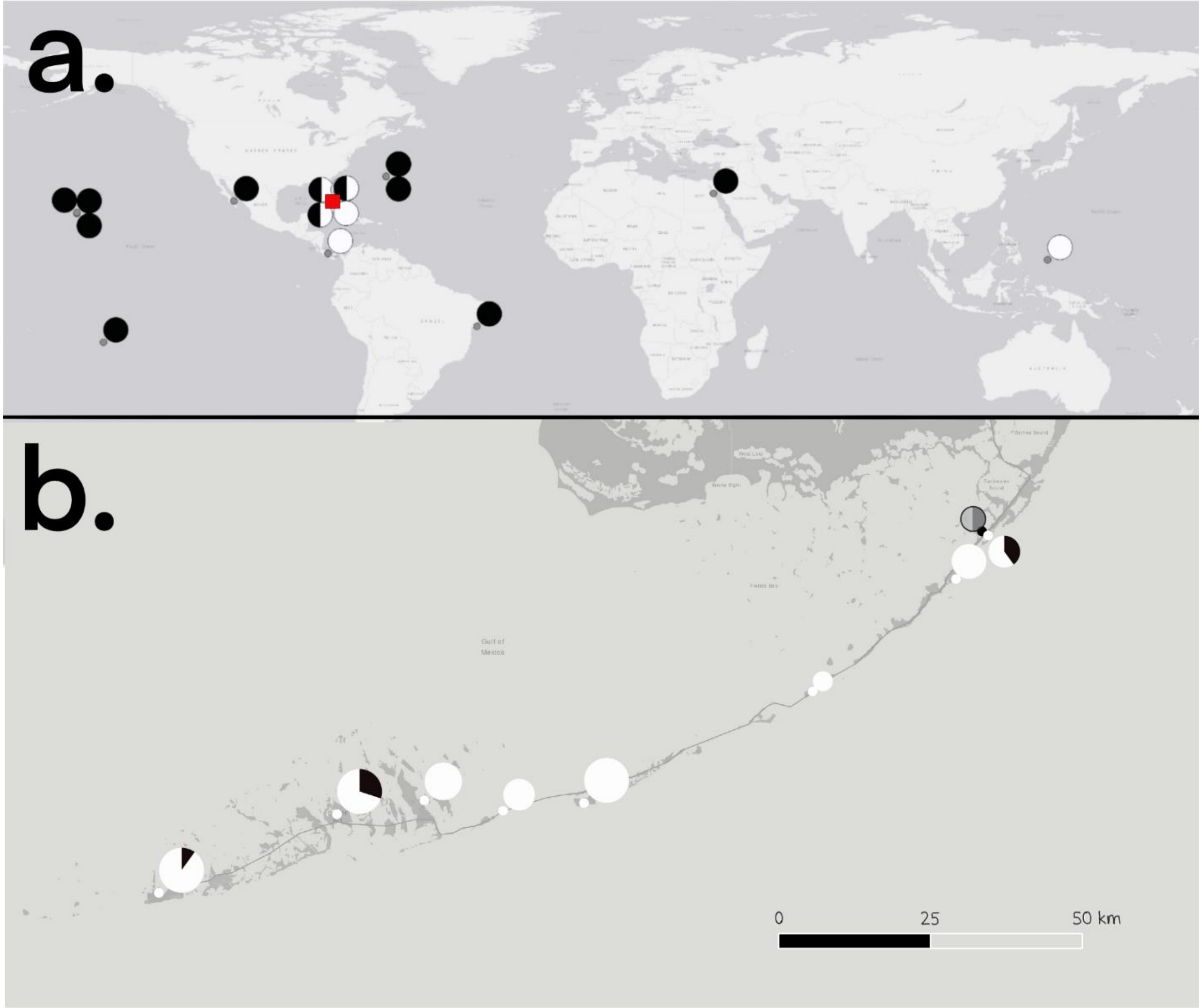

Figure 4. Color and morphological variability in collected *Cassiopea* by site in the Florida Keys. “CX” for *Cassiopea xamachana* and “CA” for *Cassiopea andromeda*.

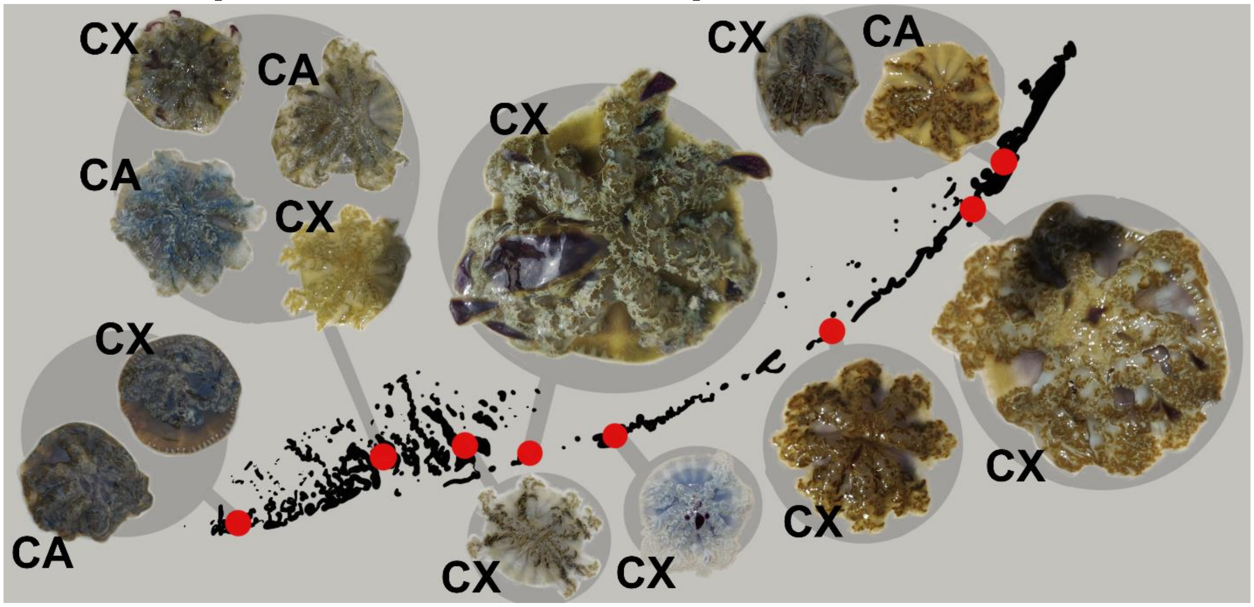

Figure 1 correctly formatted

Click here to access/download;Figure;Fig1.tif

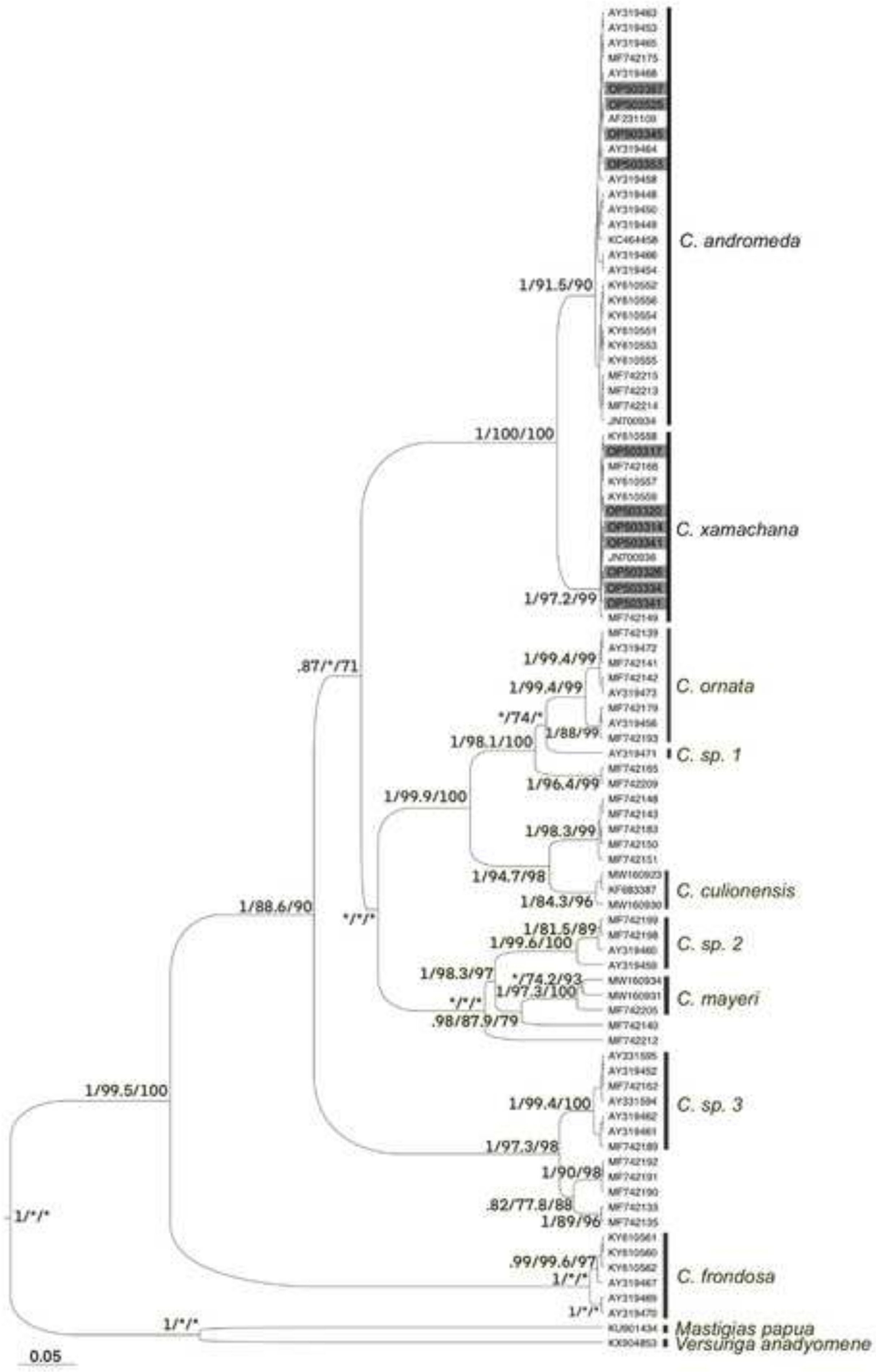

Figure 2 correctly formatted

Click here to access/download;Figure;Fig2.tif

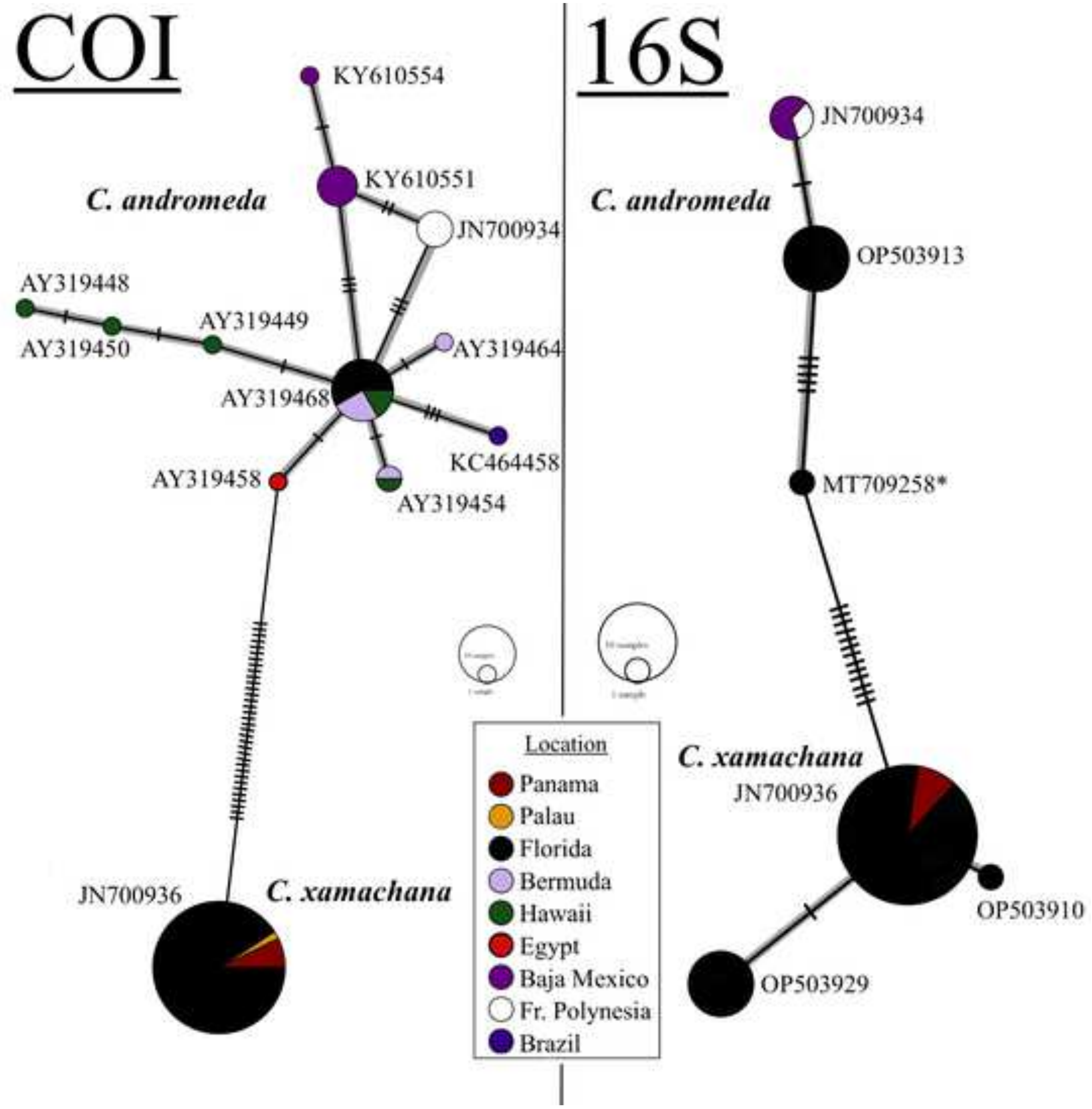

Figure 3 correctly formatted

Click here to access/download;Figure;Fig3.tif

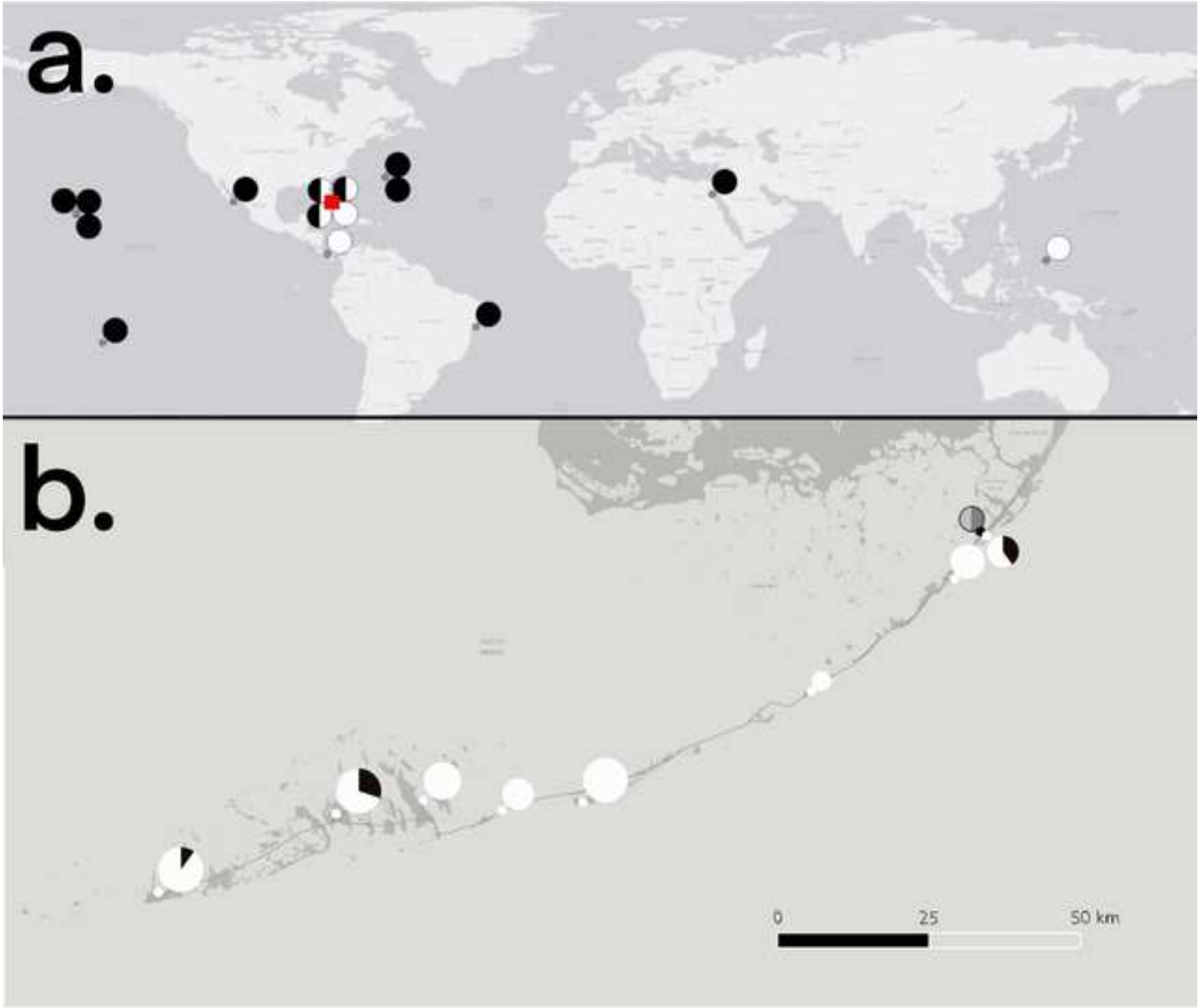

Figure 4 correctly formatted

Click here to access/download;Figure;Fig4.tif

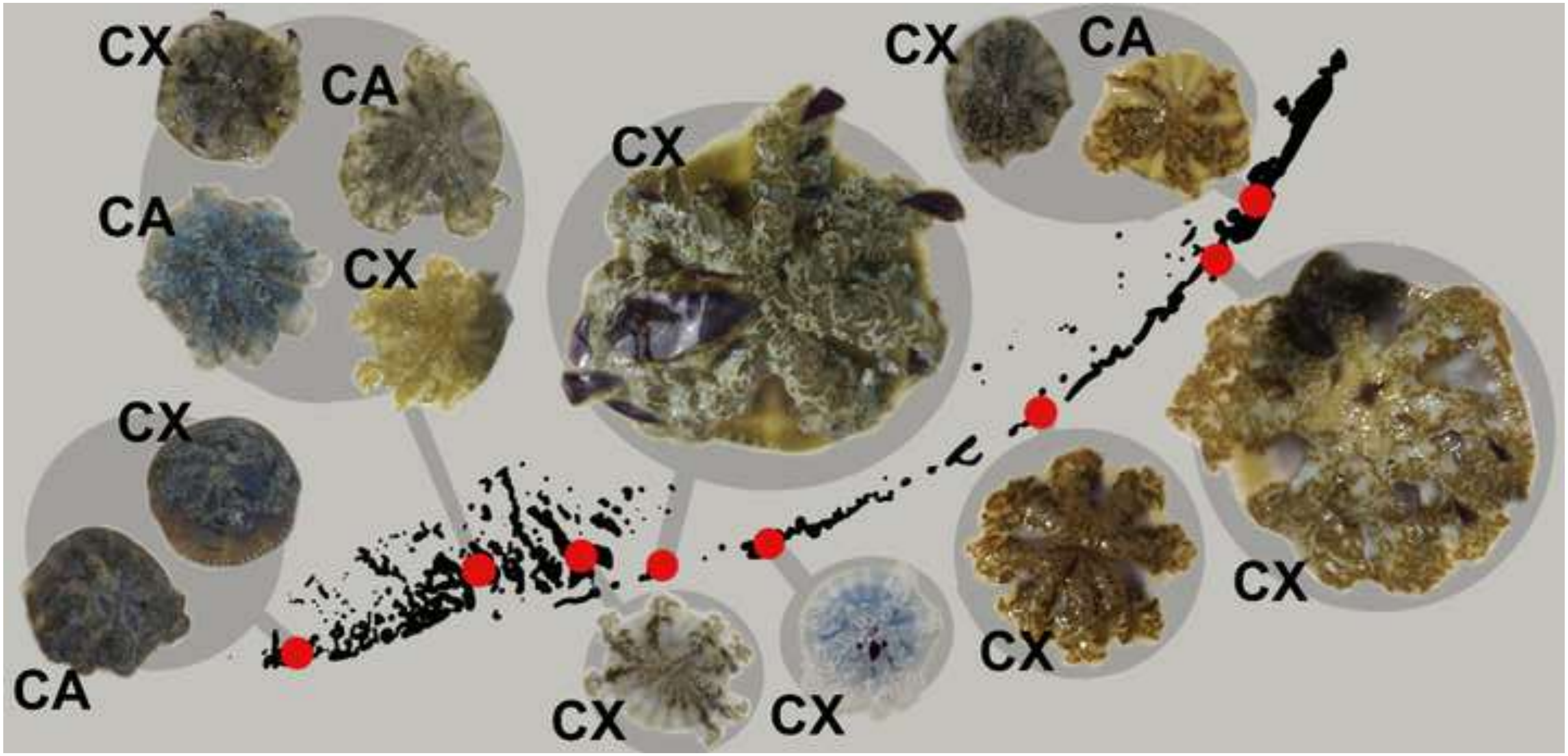

Sup Fig S1-S5

Click here to access/download
Supporting Information
Dem_PLOS_11.17.22.Sup.docx
