# Supplementary material for: Demystifying Cassiopea species identity in the Florida Keys: Cassiopea xamachana and Cassiopea andromeda coexist in shallow waters
Source: PLoS One. 2023 Mar 29;18(3):e0283441. doi: 10.1371/journal.pone.0283441 (PMC10058153; doi:10.1371/journal.pone.0283441)
Supplement: Attachment — Submitted filename: Response to Reviewers.docx [file pone.0283441.s003.docx]

Dear editor and reviewers,

Thank you for reviewing this manuscript, your thoughtful comments have improved the text substantially. In this revised draft we have made the following high-level changes to the manuscript overall:

1. The manuscript formatting, figures, etc. have been revised to comply with journal guidelines.
2. The abstract, results and discussion section have been reworked to emphasize the lack of clarity that still exists on the *C. andromeda/C. xamachana* species boundary, and the necessity for more ecological and morphological analysis of the medusae.

Specific line edit responses are listed below. We hope that you find the revisions in line with expectations.

Thank you again,

KM Muffett

Journal Requirement Compliance:

1. Please ensure that your manuscript meets PLOS ONE's style requirements, including those for file naming.

This has been corrected as specified.

2. In your Methods section, please provide additional information regarding the permits you obtained for the work. Please ensure you have included the full name of the authority that approved the field site access and, if no permits were required, a brief statement explaining why.

Permitting information has been added to the text.

3. We note that you have provided funding information that is not currently declared in your Funding Statement. However, funding information should not appear in the Acknowledgments section or other areas of your manuscript. We will only publish funding information present in the Funding Statement section of the online submission form.

Funding has been removed from the acknowledgement section and corrected. Please replace funding statement with:

This work was supported by a Texas Sea Grant to author KMM. This work was also supported by Texas A&M University Galveston. The funders had no role in study design, data collection and analysis, decision to publish, or preparation of the manuscript.

4. Please amend either the title on the online submission form (via Edit Submission) or the title in the manuscript so that they are identical.

Title has been amended.

5. Your ethics statement should only appear in the Methods section of your manuscript. If your ethics statement is written in any section besides the Methods, please move it to the Methods section and delete it from any other section. Please ensure that your ethics statement is included in your manuscript, as the ethics statement entered into the online submission form will not be published alongside your manuscript.

This has been corrected as specified.

6. We note that Figures 3 and S1 in your submission contain [map/satellite] images which may be copyrighted.

Figure three has been revised to include only public domain USGS maps. Fig S1 has been removed as unnecessary.

7. Please include a separate caption for each figure in your main manuscript.

This has been corrected as specified.

8. Please include captions for your Supporting Information files at the end of your manuscript, and update any in-text citations to match accordingly. Please see our Supporting Information guidelines for more information: <http://journals.plos.org/plosone/s/supporting-information>.

This has been corrected as specified.

9. Please review your reference list to ensure that it is complete and correct. If you have cited papers that have been retracted, please include the rationale for doing so in the manuscript text, or remove these references and replace them with relevant current references. Any changes to the reference list should be mentioned in the rebuttal letter that accompanies your revised manuscript. If you need to cite a retracted article, indicate the article’s retracted status in the References list and also include a citation and full reference for the retraction notice.

Reference list has been revised and updated to match journal guidelines.

Reviewer #1:

Dear authors,
The topic of your paper is very interesting and the paper opens a lot of questions on the species identification in that location where two different species coexist as well as past taxonomic misidentifications. All presented results seem to support the conclusion, even if sometimes they are a little bit confusing as stated along the manuscript file pdf attached.
Thank you for your kind words and your support of this manuscript.

1. Line 14: Is *Cassiopea* present beyond the Florida Keys?

Cassiopea extends beyond the Florida Keys into southern Florida with limited accounts on the panhandle (northern Fl).

2. Line 68: Are S1 and S2 supplementary figures?

S1 and S2 are supplementary figures, a description of all supplementary figures have been added at the end of the text.

3. Line 78: Why were there fewer 16S and 28S genes sequenced?

We identified clades with the 16 dataset, and selected representatives of each clade for further 28S sequencing. This allowed for the most cost/time effective approach. However, we also encountered difficulties in amplifying the 28S sequences. This resulted in having fewer 28S sequences than 16S.

4. Line 85: How did you assign sequence identity?

Sequence identity was assigned through agreement with either the published Florida Keys *C. xamachana* genome or French Polynesian *C. andromeda* mitogenome. This has now been specified in the text.

5. Move long table

Unfortunately, table one positioning must follow the first paragraph it is mentioned to be in line with journal guidelines.

6. How many sequences are included in the combined 16S and COI dataset? Did you use this to build fig 1?

The combined COI and 16S dataset includes 89 COI and 22 16S sequences. All sequences are described in the “Combined *COI* and *16S* *Cassiopea* Phylogeny Dataset” section of the methodology. Figure 1’s caption has been updated to make its provenance clearer.

7. Results section clarity

We have endeavored to improve the clarity of the results section.

8. Line 136: Did you calculate the divergence between species?

Yes, specified to clarify that this is in reference to COI divergence.

9. Line 152: Cite figure 2 before fig 3, check figure references in text

All figures have been reorganized and figure references have been checked and corrected.

10. Move speculative sentences to the discussion.

This has been corrected as specified.

11. Line 227: Rephrase statement on Cassiopea COI identity.

The statement as presented is correct, within Cassiopea xamachana COI sequences there was no nucleotide diversity.

12. Line 244: This sentence is unclear, rephrase

This has been rephased for clarity.

13. Add comments on unassigned Cassiopea sequences from fig 1 in discussion section

This has been corrected as specified.

14. Check Gamero-Mora 2022 citation, Ohdera 2019 and Deidun 2018 citation

This has been corrected as specified.

Reviewer #2

This is a very interesting paper about identifying species of *Cassiopea* in Florida. The study highlights the cryptic nature of Cassiopea and the importance of using molecular work for species identification. Additionally, it confirms that multiple species of *Cassiopea* can co-exist in the same area, and so location should not be used as a factor to identify species.

Overall, the paper is well written, and represents the results in a way that is easy to understand and interpret. However, I do have a couple of comments and questions that should be addressed in the paper. Mainly, the authors conclude that *C. andromeda* and *C. xamachana* are different species based off genetics and morphology. However, the authors also state that they did not complete any detailed morphological comparison between the two species. To be able to definitively conclude that there are two species occurring, especially as they so closely related, a detailed morphological comparison needs to be done before making such a conclusion. As a result, the authors need to tone back their conclusion that they are definitely different species and include another paragraph in the discussion discussing this further, along with strengthening their argument about why the authors concluded that there are two species based on genetics.

Thank you for your support and constructive criticism of the manuscript. We have revised claims on species identity to bring them more into line with the uncertain nature of the data, and added a call for detailed morphological and ecological study in the discussion section.

1. Line 10: “upset multiple times” is unclear terminology. “Confused” may be a better term to use here

“Upset” has been replaced with “revised”.

2. Line 26: “sampled broadly but shallowly” is confusing terminology. Do the authors mean shallow depth here? What depths do they define as shallow? These references refer to Cassiopea, which are typically found in shallow environments. Aurelia have been collected at depths of up to 18m though and so a clearer way of describing this could be close to shore.

Thank you, “broadly but shallowly” here meant sampled across a large geographic region but with limited specimen numbers at each location. This has been replaced so as to make the text clearer.

3. Line 30: What other scyphozoan lineages?

Possible additionally lineages may be near-shore invaders like *Phyllorhiza* spp., outside of scyphozoa, genera like *Turritopsis* also have a history defined by cryptic and near-cryptic invasion. Without detailed study of the variation within coastal lineages like *Phyllorhiza*, with so many undersampled locations, diversity is impossible to ascertain.

4. Line 44: Is C. xamachana morphologically distinct from C. andromeda? They have often been confused taxonomically because they have similar morphological characteristics. Additionally, this is conflicting with the rest of the paper.

Thank you for suggesting I clarify this. While the descriptions are certainly distinct, the traits discussed are highly variable. I have now added this line and switched the less familiar “ecotype” with the more familiar “morphotype”.

5. Line 61: I think the authors need to clarify that they did not look at deeper depths, where C. frondosa are known to occur. Cite the Fitt et al. 2021 paper.

This has now been clarified.

6. Line 69: What was measured? Their diameter?

The diameter was measured, this has now been clarified in text.

7. Line 70: why were the samples put in ethanol rather than formalin for morphological analysis?

Preserved samples were not originally intended for morphological analysis, this was an addition made later.

8. Line 136: Need references for previously published mitogenomes

This has been corrected as specified.

9. Line 152: There are other sequences of C. xamachana on Genbank that are not in the Caribbean. Please see Stampar et al (2021) The puzzling occurrence of the upside-down jellyfish Cassiopea (Cnidaria: Scyphozoa) along the Brazilian coast: a result of several invasion events? And Gamero-Mora (2019) Regenerative capacity of the upside-down jellyfish Cassiopea xamachana.

Thank you. I had originally excluded laboratory specimens, however, seeing the provenance of the colony I will now update the paper to reflect that *C. xamachana* is found in Brazil as well.

10. Line 188: How shallow?

Corrected. Specified as under 1m depth.

11. Line 190: How did the authors measure the density for each site? This is not included in the methods.

A m2 was marked and individuals within that area were counted. This has now been added to the collection section of the methods.

12. Line 194: Deeper sampling? What depths do they mean by this? Cassiopea frondosa has been sampled at deeper depths within the area so this needs to be clarified

Deeper here meant “greater N”. This has been replaced.

13. Line 201: These stats for the difference between bell diameters needs to be included in the methods

This has been corrected as suggested.

14. Line 204: If no comparative morphological analysis could be done, line 198 needs to be revised. The authors shouldn’t state that there were no morphological differences between the two species if no detailed comparison was done

This has been corrected as suggested.

15. Line 211: A bit more detail needs to be provided here. Did the colour vary between species as well as between sites?:

Coloration of *C. andromeda* mitotype individuals was similar to C. xamachana individuals at sites where they cooccurred. A note not this effect has been added.

16. Line 214: Remove “dramatically”

This has been corrected as suggested.

17. Line 216: Remove “only”

This has been corrected as suggested.

18. Line 221: Hard to conclude that *C. andromeda* and *C. xamachana* are different species when they are in the same monophyletic group and this study did not do any morphological comparisons, but state that there was no morphological difference between species. This sentence needs to be revised as such as I don’t believe the authors can confidently state that C. andromeda and C. xamachana are different species without a proper morphological comparison. If the authors are going to state there are no morphological differences between the species, references need to be included to support this.
    Expanding on above, I think the authors need to include a paragraph discussing how a proper morphological comparison needs to be done to support their findings that genetics shows that two species are occurring in Florida. AND Line 265: Again, the authors state that there were no morphological differences between the two species, but have also acknowledged that they didn’t do a detailed morphological comparison.

We have added text to the discussion calling for morphological and ecological study of mixed populations to determine whether there are indeed differences, we have also walked back claims about morphology and increased emphasis on the lack of clarity in the nature of the species boundary (including presence/absence).

Reviewer #3: The manuscript is well written, clear and easy to understand. Below are some specific observations/ suggestions.

1. Line 15 – Scientific names are commonly written out in full when they first appear in the abstract.

This has been corrected as suggested.

2. Lines 31, 42, 94, etc. – The names are again written out in full when they first appear after the abstract and are then abbreviated upon further use.

This has been corrected as suggested.

3. Line 43 -- Ecotype could replaced by species or taxon.

Replaced by morphotype, as it is not clear that the foundational traits on which they were differentiated are distinctive between groups and often vary within *C. xamachana* and *C. andromeda* (e.g. Color)

4. Line 65 – “through agreement >98% to either genome.”, which is the reference to those genomes?

This has been corrected as suggested.

5. Line 112 – For the multi-gene analysis (16S+COI), do the authors allowed individual models for the different genomic loci (genes or codon positions)? Why does the 28S dataset was not included in the combined analysis for tree generation?

Individual models were used as laid out in the paragraph starting with “Models for all COI codons” (*COI* codon position 1: TN93+I, *COI* codon position 2: TN93+I, *COI* codon position 3: TN93+G+I, *16S*: GTR+G+I). With such a limited comparative dataset (too few *Cassiopea* with previously published 28S sequences) addending it to the COI and 16S seemed inadvisable.

6. Lines 140, 154, 160, 228, etc. – Mastigias papua, Versuriga anadyomene, Cassiopea andromeda, Cassiopea xamachana can be abbreviated (they were written out in full previously).

This has been corrected as suggested.

7. Some scientific names appear abbreviated at the beginning of sentences (e.g., line 176) while others were written out in full at the beginning of sentences (e.g., line 162), I suggest homogenizing the format.

This has been corrected as suggested.

8. Figure 3b could be improved by adding information about latitude and longitude (e.g., by adding, to the figure, an upper layer with a coordinate grid) and using a bigger font size for “Gulf of Mexico” and “Florida Keys ”

This has been corrected as suggested.

9. Figure 4 could be improved by coloring with two colors the circles that indicate the locations where both species were recorded (black and white as in figure three).

This has been corrected as suggested.
